# Targeting CDC7 potentiates ATR-CHK1 signaling inhibition through induction of DNA replication stress in liver cancer

**DOI:** 10.1186/s13073-021-00981-0

**Published:** 2021-10-18

**Authors:** Yuchen Guo, Jun Wang, Bente Benedict, Chen Yang, Frank van Gemert, Xuhui Ma, Dongmei Gao, Hui Wang, Shu Zhang, Cor Lieftink, Roderick L. Beijersbergen, Hein te Riele, Xiaohang Qiao, Qiang Gao, Chong Sun, Wenxin Qin, René Bernards, Cun Wang

**Affiliations:** 1 State Key Laboratory of Oncogenes and Related Genes, Shanghai Cancer Institute & Department of Liver Surgery, Renji Hospital, Shanghai Jiao Tong University School of Medicine, Shanghai, China; 2 Division of Molecular Carcinogenesis, Oncode Institute, The Netherlands Cancer Institute, Plesmanlaan 121, 1066 CX Amsterdam, The Netherlands; 3 Division of Tumour Biology and Immunology, The Netherlands Cancer Institute, Plesmanlaan 121, 1066 CX Amsterdam, The Netherlands; 4 Liver Cancer Institute, Zhongshan Hospital, Fudan University, Key Laboratory of Carcinogenesis and Cancer Invasion, Ministry of Education, Shanghai, China; 5 Immune Regulation in Cancer Group, German Cancer Research Center, D-69120 Heidelberg, Germany

**Keywords:** Hepatocellular carcinoma, ATR-CHK1 signaling, Replication stress, Cell division cycle 7

## Abstract

**Background:**

Liver cancer is one of the most commonly diagnosed cancers and the fourth leading cause of cancer-related death worldwide. Broad-spectrum kinase inhibitors like sorafenib and lenvatinib provide only modest survival benefit to patients with hepatocellular carcinoma (HCC). This study aims to identify novel therapeutic strategies for HCC patients.

**Methods:**

Integrated bioinformatics analyses and a non-biased CRISPR loss of function genetic screen were performed to identify potential therapeutic targets for HCC cells. Whole-transcriptome sequencing (RNA-Seq) and time-lapse live imaging were performed to explore the mechanisms of the synergy between CDC7 inhibition and ATR or CHK1 inhibitors in HCC cells. Multiple in vitro and in vivo assays were used to validate the synergistic effects.

**Results:**

Through integrated bioinformatics analyses using the Cancer Dependency Map and the TCGA database, we identified ATR-CHK1 signaling as a therapeutic target for liver cancer. Pharmacological inhibition of ATR or CHK1 leads to robust proliferation inhibition in liver cancer cells having a high basal level of replication stress. For liver cancer cells that are resistant to ATR or CHK1 inhibition, treatment with CDC7 inhibitors induces strong DNA replication stress and consequently such drugs show striking synergy with ATR or CHK1 inhibitors. The synergy between ATR-CHK1 inhibition and CDC7 inhibition probably derives from abnormalities in mitosis inducing mitotic catastrophe.

**Conclusions:**

Our data highlights the potential of targeting ATR-CHK1 signaling, either alone or in combination with CDC7 inhibition, for the treatment of liver cancer.

**Supplementary Information:**

The online version contains supplementary material available at 10.1186/s13073-021-00981-0.

## Background

Liver cancer poses a significant threat to human health, as it is one of the most lethal cancer types with a 5-year survival of only 18% [1]. The histological subtype of the majority of liver cancer cases is hepatocellular carcinoma (HCC). Sorafenib, a multi-kinase inhibitor approved by US Food and Drug Administration (FDA) as the standard therapy for advanced HCC patients in 2007, only provides less than 3-months benefit in median overall survival [2]. Many systematic therapies, including lenvatinib [3], regorafenib [4], cabozantinib [5], pembrolizumab [6] and nivolumab [7, 8], have currently been approved by FDA for the treatment of late-stage or unresectable HCC patients. Most of them, however, only provide limited survival benefit [9, 10]. The recent IMbrave150 trial indicates that the combination of atezolizumab and bevacizumab resulted in better survival outcomes than sorafenib monotherapy, providing a confirmed objective response rate of 27.3% [11]. Despite this progress, further investigations of novel therapeutic strategies are urgently required to counter this lethal disease.

ATR and CHK1 were identified as potential therapeutic targets for cancer. ATR-CHK1 signaling is a key regulator of the DNA damage response (DDR) involved in sensing DNA replication stress due to oncogene activation or impairment of G1 checkpoint regulation [12, 13]. Given the broad role of ATR-CHK1 signaling in the DDR, development of ATR and CHK1 inhibitors has received considerable attention from oncology drug developers. Although ATR and CHK1 inhibitors have shown anti-tumor effects as monotherapy in preclinical studies, the clinical effects of these drugs most likely will be dependent on using the right combination therapies and biomarkers-guided patient classification [12, 14–20]. It has been demonstrated that ATR and CHK1 inhibitors can potentiate the efficacy of genotoxic chemotherapies, such as doxorubicin, irinotecan, and gemcitabine, which are strong inducers of DNA damage [12, 14–16]. Several studies have suggested that tumor cells with high levels of DNA replication stress (overexpressing replication stress-inducing oncogenes such as *RAS*, *CCNE1* or *MYC*), genetic deficiencies in *TP53* or *ATM*, or defects in homologous recombination, will likely be more vulnerable to ATR inhibitors [17–20]. In liver cancer, however, despite some preliminary findings [21–23], the therapeutic benefit of ATR and CHK1 inhibitors remain to be explored.

The cell division cycle 7 (CDC7) protein plays key roles in DNA replication initiation, the S-phase checkpoint, and M-phase completion [24]. *CDC7* expression is upregulated in HCC tumor tissues relative to paired non-tumor tissues, which provides a potential therapeutic window for cancer treatment [25]. Bioavailable selective CDC7 inhibitors (TAK-931 and LY3143921) [26, 27] have recently entered phase I clinical trials in patients with advanced solid tumors. It has been reported that inhibition of CDC7 significantly reduces replication fork initiation and then induces replication stress [28, 29].

Here, we use integrated bioinformatics and functional genetics approaches to study the therapeutic options for the use of ATR or CHK1 inhibitors in liver cancer. Our study provides a potential classification and treatment strategy for liver cancer patients based on DNA replication stress levels of tumor tissues.

## Methods

### Human cell lines

The human HCC cell lines, Hep3B, Huh7, SNU182, PLC/PRF/5, SNU398, HepG2, Huh6, and SNU449 were provided by Erasmus University (Rotterdam, Netherlands). MHCC97H was provided by the Liver Cancer Institute of Zhongshan Hospital (Shanghai, China). HCC cells were cultured in Dulbecco’s Modified Eagle Medium (DMEM) with 10% fetal bovine serum, glutamine, and penicillin/streptomycin (Gibco) at 37 °C/5% CO_2_. Mycoplasma contamination was excluded via a PCR-based method. The identities of all the cell lines were confirmed by short tandem repeat (STR) profiling.

### Compounds and antibodies

AZD6738 (S7693), MK-8776 (S2735), XL413 (S7547), LY3177833 (206762), BAY-1895344 (S8666), LY2606368 (S7178), Cisplatin (S1166), and Z-VAD-FMK (S7023) were purchased from Selleck Chemicals. XL413 (205768) and AZD6738 (206114) were purchased from MedKoo Bioscience. TAK-931 (CT-TAK931) was purchased from Chemietek. Antibodies against HSP90 (sc-7947, sc-13119), Cyclin B1 (sc-245), ATR (sc-515173), and CHK1 (sc-8408) were purchased from Santa Cruz Biotechnology. Antibody against γH2AX (05-636-AF647) was purchased from Merck Millipore. Antibodies against p-MCM2 (ab109133, ab133243) and MCM2 (ab4461) were purchased from Abcam. Antibody against β-actin (66009-1-Ig) was purchased from Proteintech. Antibodies against p-ATR (Thr1989) (30632) and p-CHK1 (Ser345) (2348 T) were purchased from Cell Signaling Technology.

### Pooled CRISPR screen and data processing

For the design of the kinome CRISPR library, 5971 gRNAs targeting 504 human kinases, 10 essential genes, and 50 non-targeting gRNAs were selected (Additional file 1: Table S1). Oligos with gRNA sequences flanked by adapters were ordered from CustomArray (Bothell, Washington, USA) and cloned as a pool by GIBSON assembly in LentiCRISPRv2.1 [30]. The kinome CRISPR library was introduced into MHCC97H cells by lentiviral transduction. Cells stably expressing gRNA were cultured for 14 days. The abundance of each gRNA in the pooled samples was determined by Illumina deep sequencing. Single-end reads were trimmed and quality-filtered and then matched against sgRNA sequences from the kinome CRISPR library. Subsequently, read counts of sgRNAs were normalized against total read counts across all samples. For each sgRNA, the fold change value for enrichment was calculated between the T14 (cultured for 14 days) group and T0 group.

### Protein lysate preparation and western blots

Cells were washed with phosphate-buffered saline (PBS) and lysed with RIPA buffer supplemented with Complete Protease Inhibitor (Roche) and Phosphatase Inhibitor Cocktails II and III (Sigma). All lysates were freshly prepared and processed with Novex NuPAGE Gel Electrophoresis Systems (Invitrogen).

### Cell proliferation assays

Cells were seeded into six-well plates (1.5–3 × 10^4^ cells per well) and cultured in the presence of drugs as indicated. For each cell line, cells cultured at different conditions were fixed with 4% paraformaldehyde (in PBS) at the same time. Afterwards, cells were stained with 0.1% crystal violet (in water).

### Incucyte cell proliferation assay and apoptosis assay

Cells were cultured and seeded into 96-well plates at a density of 1000–1500 cells per well. Twenty-four hours later, drugs were added at indicated concentrations. Cells were imaged every 4 h in IncuCyte ZOOM (Essen Bioscience). Phase-contrast images were collected and analyzed to detect cell proliferation based on cell confluence. For cell apoptosis, caspase-3/7 green apoptosis assay reagent was also added to culture medium and cell apoptosis was analyzed based on green fluorescent staining of apoptotic cells. For each condition, at least three replicates (50 cells/field) were analyzed.

### RNA sequencing and data processing

PLC/PRF/5 cells were cultured in the presence or absence of 10 μM XL413 for 96 h. Total RNA was extracted using the Trizol reagent (Invitrogen), and the library was prepared using TruSeq RNA sample prep kit according to the manufacturer’s protocol (Illumina). For data analysis, raw sequencing reads were mapped to the human genome (GRCh38) using STAR (version 2.4.2 g1) [31]. Then, gene-level read counts were generated using featureCounts from the subRead package with default settings [32].

### Neutral comet assay

Cells were harvested and embedded in 1% low-gelling-temperature agarose (Sigma-Aldrich). Cell suspension was used to make gels onto comet assay slides (Trevigen). Cells in the agarose gels were lysed at 37 °C in lysis buffer (2% sarkosyl, 0.5 M Na_2_EDTA, and 0.5 mg/ml Proteinase K) overnight. Subsequently, slides were washed three times for 30 min at room temperature in electrophoresis buffer (90 mM Tris-HCl pH = 8.5, 90 mM Boric Acid and 2 mM Na_2_EDTA). Electrophoresis was performed for 25 min at 20 V in electrophoresis buffer. Afterwards, slides were washed once with MQ and DNA was stained using 2.5 μg/ml propidium iodide (PI) in MQ. Individual comets were imaged with Zeiss AxioObserver Z1 inverted microscope. Tail moment of individual comets was assessed using the CASP software. For each condition, at least 50 cells were analyzed.

### DNA fiber analysis

Cells were pulse labeled with 25 μM CldU followed by 250 μM IdU for 20–45 min each. Labeled cells were trypsinized, lysed in spreading buffer (200 mM Tris-Hcl pH 7.4, 50 mM EDTA, and 0.5% SDS) and spread on microscope slide (Menzel-Gläser, Superfrost). DNA fibers were fixed on slides using 3:1 methanol: acidic acid. Slides were treated with 2.5 M HCl for 1 h and 15 min to denature DNA followed by 1 h incubation in blocking buffer (PBS, 1% BSA, 0.1% Tween20) to block background staining. For detection of CldU and IdU, slides were incubated for 1 h with rat-anti-Brdu (Clone BU1/75, Abcam; 1:500) and mouse-anti-BrdU (clone B44, Becton Diskinson; 1:750), respectively. Subsequently, slides were fixed with 4% paraformaldehyde for 10 min and incubated with Alexa 488-labeled goat-anti-mouse and Alexa 555-labeled goat-anti-rat (Molecular probes; 1:500) for 1 h and 30 min. DNA fibers were imaged with the Zeiss AxioObserver Z1 inverted microscope using a 63x objective equipped with a Hamamatsu ORCA AG Black and White CCD camera. Replication tracks lengths were analyzed using ImageJ software and the conversion factor 1 μm = 2.59 kb was used.

### Time-lapse live imaging

To allow visualization of chromosomes, cells were transduced with a histone H2B-GFP (LV-GFP, Addgene plasmid#25999). Cells were then plated 24 h before starting the microscope acquisition. XL413 (10 μM), AZD6738 (1.25 μM), and MK-8776 (2.5 μM) were added in the medium 1 h before starting the movie. Cells were filmed over 96 h and pictures were taken every 8 min. For each condition filmed, 5 different fields were selected. In each field, we randomly chose and followed cells entering in mitosis (nuclear envelope breakdown, NEBD, was used as indicator of mitotic division onset).

### Xenografts

Animals were housed in micro-isolator cages of dimensions 30.5 cm × 19 cm × 14 cm, including a wire rack in the cage for holding food and a water bottle. Animals were housed on a 12-h light/dark cycle. A carbon dioxide (CO2) euthanasia method was applied to mice. All animals were manipulated and housed according to protocols approved by the Shanghai Medical Experimental Animal Care Commission and Shanghai Cancer Institute. Huh7 and MHCC97H cells (1 × 10^7^ cells per mouse) were injected subcutaneously into the right posterior flanks of 6-week-old BALB/c nude mice (male, 8-9 mice per group). Tumor volume based on caliper measurements was calculated by the modified ellipsoidal formula: tumor volume = 1/2 length × width^2^. After tumor establishment, mice were randomly assigned to 5 days/week treatment with vehicle, or AZD6738 (50 mg/kg, oral gavage). For combination treatment assay, PLC/PRF/5 cells (1 × 10^7^ cells per mouse) were injected subcutaneously into the right posterior flanks of 6-week-old BALB/c nude mice (male, 8 mice per group). Mice were randomly assigned to treatment 3 days/week with vehicle, XL413 (50 mg/kg, oral gavage), AZD6738 (50 mg/kg, oral gavage), or a drug combination in which each compound was administered at the same dose and schedule as single agent.

### Immunohistochemical staining

Formalin-fixed paraffin-embedded samples were obtained from xenograft tumors and then probed with antibodies against γH2AX (#9718, Cell Signaling Technology). Following incubation with the primary antibodies, positive cells were visualized using DAB+ as a chromogen.

### Included public datasets

Three clinical cohorts, including two RNAseq-based cohorts (CHCC and TCGA-LIHC) and one microarray-based cohort (GSE14520) were utilized for conducting clinical analysis [33–35]. Fragments per kilobase per million reads (FPKM) normalized transcriptome of CHCC cohort was downloaded from NODE (www.biosino.org/node). Gene expression data (raw counts) of LIHC cohort was obtained from TCGA website (portal.gdc.cancer.gov/repository) [34]. Expression data of GSE14520 cohort was assessed from the Gene Expression Omnibus (GEO) (www.ncbi.nlm.nih.gov/geo/) [36]. For sequencing data, raw counts, or FPKM normalized expression data were transformed into transcripts per kilobase million (TPM) values for subsequent analysis. For microarray data, robust multi-array average (RMA) method was used for normalization. Clinical data of samples from CHCC and GSE14520 cohorts were obtained from corresponding supplementary files [33, 35]; clinical data of TCGA cohort were downloaded from the TCGA Pan-Cancer Clinical Data Resource (TCGA-CDR) [37]. Drug sensitivity and molecular data of hundreds of cancer cell lines (CCLs) were obtained from the genomics of drug sensitivity in cancer (GDSC) (released October 2019, cancerrxgene.org) [38] and PRISM Repurposing dataset (19Q4, released December 2019, depmap.org/portal/prism/) [39]. The area under the dose-response curve (AUC) values in these datasets were used as a measure of drug sensitivity, and lower values indicate increased sensitivity to treatment. Gene dependency data was achieved from the dependency map (DepMap) portal (depmap.org/portal/) [40]. CERES score is taken as a measure of the gene dependency, and a lower CERES score indicates a higher likelihood that the gene is essential for cell growth and survival [40].

### Bioinformatics analysis

Th replication stress gene set (c2.cp.reactome.v7.4) came from the Molecular Signatures Database (MSigDB), which included 37 replication stress-associated genes [41]. For Gene set enrichment analysis (GSEA), the fold change (FC) of each gene between subclasses was firstly calculated, and input genes were ranked in descending order according to the FC values. GSEA was then performed using GSEA software or clusterProfiler R package based on the replication stress gene set [42]. For single sample gene set enrichment analysis (ssGSEA), ssGSEA scores for each sample were computed through GSVA R package with default parameters based on the replication stress gene set [43]. ConsensusClusterPlus R package was utilized for k-means clustering [44]. Clustering was performed based on 37 replication stress-associated genes using Euclidean distance with 1000 iterations and 80% of sample resampling.

### Statistical analysis

All the computational analyses and graphical visualization were performed with Prism (Graph Pad Prism v7) and R statistical software (Cran R project R program v3.6.0). Comparison of a continuous variable in two or more than two groups was performed using either a parametric test (Student’s *t* test or analysis of variance) or a nonparametric test (Wilcoxon rank-sum test or Kruskal–Wallis test). Correlation between two continuous variables was measured by either Pearson’s *r* correlation or Spearman’s rank-order correlation. Survival analysis was carried out using Kaplan–Meier methods and the log-rank test was used to determine the statistical significance of differences. A two-tailed *P* < 0.05 was considered significant unless indicated otherwise.

## Results

### ATR and CHK1 are potential therapeutic targets for liver cancer

**Fig. 1:**
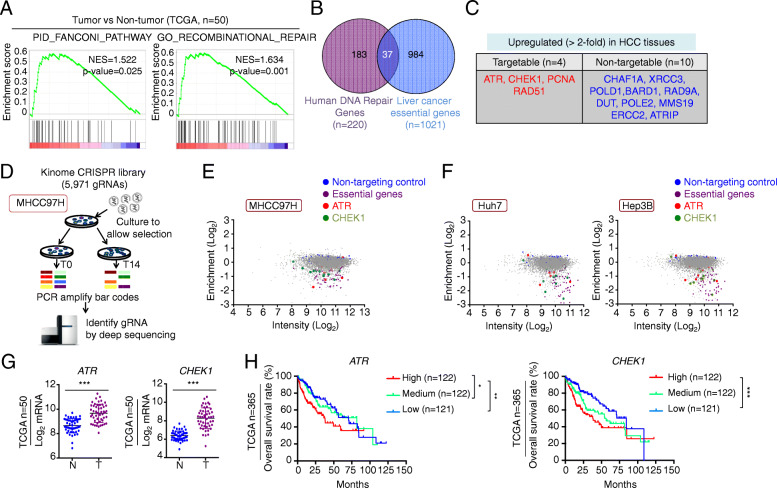
ATR and CHK1 are potential therapeutic targets for HCC. **a** Gene Set Enrichment Analysis (GSEA) indicating that FANCONI pathway and recombinational repair gene sets are enriched in HCC tissues compared with paired non-tumor tissues from the TCGA cohort (*n* = 50). **b** Venn diagram showing the overlap of human DNA repair genes (*n* = 220) and liver cancer essential genes (*n* = 1021) derived from the Dependency Map database. **c** Among the 37 overlapping genes, four targetable and ten non-targetable genes were identified, which are upregulated in HCC tissues compared with paired non-tumor tissues from the TCGA cohort (> 2-fold, *n* = 50). **d** Schematic representation of the CRISPR-Cas9 based kinome screens performed in MHCC97H cells. MHCC97H cells were infected with a lentiviral kinome gRNA library and cultured for 14 days (T14) in three replicates. gRNA barcodes from T0 and T14 samples were recovered by PCR and analyzed by next generation sequencing. **e** Representation of the relative abundance of the gRNA barcode sequences from the kinome screens. ATR and CHEK1 were identified as high-confidence lethal genes in MHCC97H cells. The *y*-axis shows log2-fold change in abundance (ratio of gRNA frequency in T14 sample to that in T0 sample). The *x*-axis depicts the average read-count in the T0 sample. **f** Re-analysis of previous kinome screen data in Huh7 and Hep3B cells. ATR and CHEK1 were identified as high-confidence lethal genes in each cell line. **g** mRNA levels of *ATR* and *CHEK1* in tumor tissues and paired non-tumor tissues in the cohort of TCGA database (*n* = 50). **h** Kaplan-Meier curves indicate that high levels of *ATR* and *CHEK1* mRNA correlate with poor prognosis of patients with HCC in the cohort of TCGA database (*n* = 365). *P < 0.05, **P < 0.01, and ***P < 0.001

To evaluate the activity of the DDR in HCC tissues, we first performed gene set enrichment analyses on RNA-sequencing data of 50 paired tumor and non-tumor tissues from the TCGA database. Two DDR related gene sets, including FANCONI pathway and RECOMBINATIONAL REPAIR pathway, were significantly enriched in HCC tissues compared to non-tumor tissues (Fig. 1a). This suggests that targeting the DDR could be exploited as a potential treatment strategy for liver cancer. To explore genes involved in the DDR as potential drug targets in HCC therapy, we analyzed the gene dependencies of liver cancer cell lines (*n* = 22) in the DepMap public dataset. A total of 1021 genes were identified as liver cancer essential genes based on the probabilities of dependency, which are provided by DepMap public dataset (Fig. 1A and Additional file 2: Fig. S1a). A total of 220 human DNA repair genes described in previous studies [45–47] were used here (Fig. 1B and Additional file 3: Table S2). Among these 220 human DNA repair genes, 37 were liver cancer essential genes (Fig. 1B) and 14 of these 37 candidate genes were upregulated over 2-fold in primary liver cancer tissues according to the TCGA database (Fig. 1C). *ATR* and *CHEK1*, two master regulators of the DDR process with specific inhibitors already in clinical trials, were selected for further investigation (Additional file 2: Fig. S1a-b).

In parallel to these in silico studies, we performed a CRISPR-Cas9 genetic screen with a lentiviral guide RNA (gRNA) library that represents all human kinases in MHCC97H cells to find liver cancer-essential kinases (Fig. 1D). In agreement with the finding from the DepMap public dataset, we found that both *ATR* and *CHEK1* genes were required for the proliferation and survival of MHCC97H cells (Fig. 1E and Additional file 1: Table S1). We next re-analyzed our previous kinome screen data in Huh7 and Hep3B cells [25]. Here too, *ATR* and *CHEK1* were identified as high-confidence lethal genes in both cell lines (Fig. 1F and Additional file 1: Table S1). Furthermore, the levels of *ATR* and *CHEK1* mRNA were upregulated in tumor tissues compared to non-tumor tissues in the TCGA database (*n* = 50) and the GSE14520 cohort (*n* = 213) (Fig. 1G and Additional file 2: Fig. S1c). Moreover, in a TCGA cohort of 365 patients with HCC stratified by tertile cut-off values, patients with the highest levels of *ATR* or *CHEK1* mRNA in their tumors exhibited the worst survival (Fig. 1H).

**Fig. 2:**
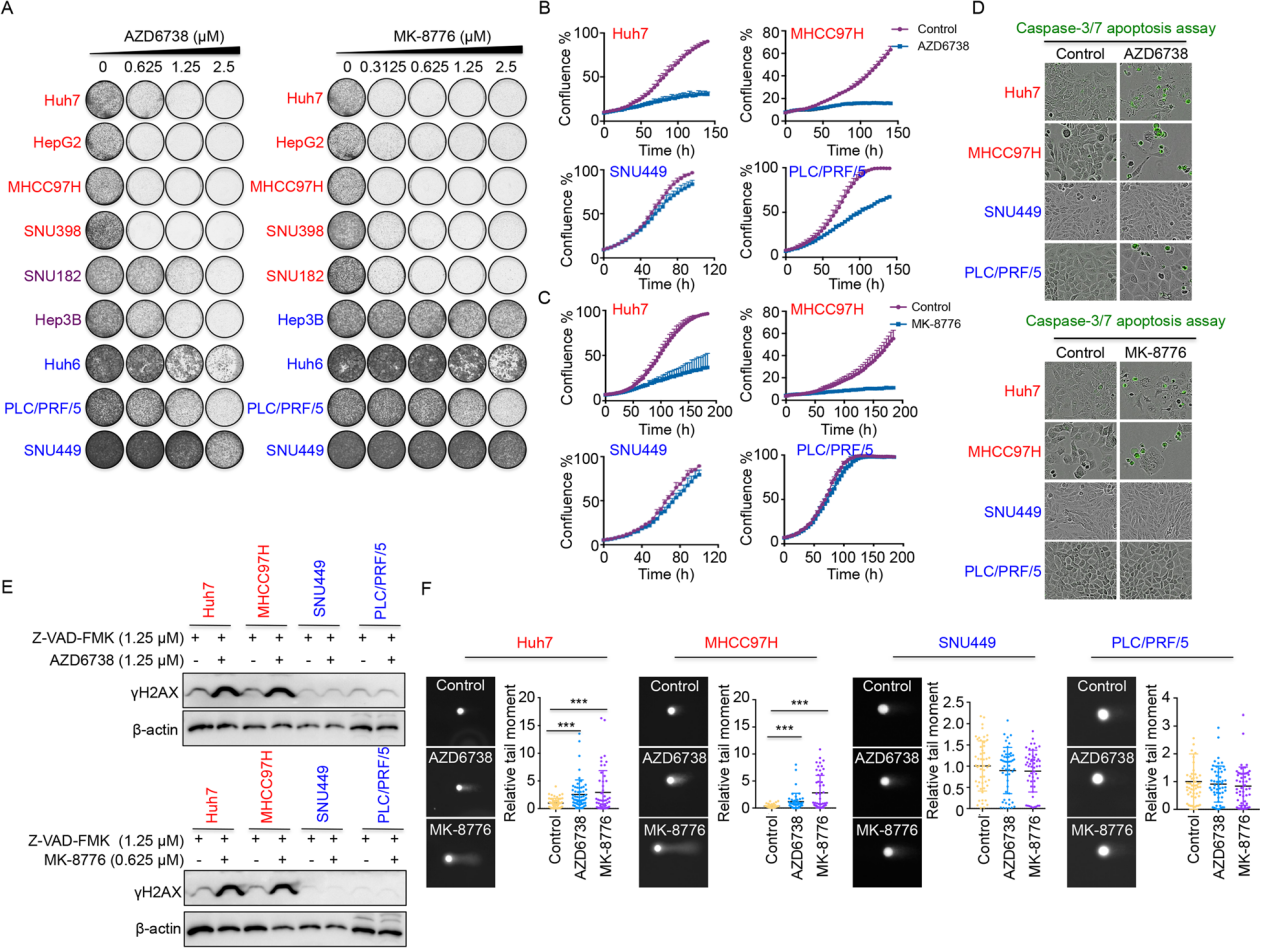
Effects of ATR and CHK1 inhibitors on HCC cells. **a** Long-term colony formation assays of a panel of liver cancer cell lines treated with increasing concentrations of AZD6738 (ATR inhibitor, left panel) or MK-8776 (CHK1 inhibitor, right panel) for 10–14 days. Cell lines indicated in red are sensitive to ATR or CHK1 inhibitors, in purple are intermediate sensitive and blue cell lines are resistant to ATR or CHK1 inhibitors. **b**, **c** IncuCyte cell proliferation assays of ATR or CHK1 inhibitors sensitive cells (Huh7 and MHCC97H) and resistant cells (SNU449 and PLC/PRF/5) in the presence or absence of 1.25 μM AZD6738 (ATR inhibitor) or 0.625 μM MK-8776 (CHK1 inhibitor) for 6–8 days. **d** Representative images of AZD6738 (upper panel) or MK-8776 (lower panel) treated HCC cells in the presence of a green fluorescent caspase-3/7 activatable dye. **e** Western blot analysis of γH2AX as a DNA damage marker in sensitive cells (Huh7 and MHCC97H) and resistant cells (SNU449 and PLC/PRF/5) in the presence or absence of 1.25 μM AZD6738 or 0.625 μM MK-8776 for 72 h. β-actin protein level served as a loading control. **f** Representative neutral-comet assay images from ATR or CHK1 inhibitors sensitive cells (Huh7 and MHCC97H) and resistant cells (SNU449 and PLC/PRF/5) treated with 1.25 μM AZD6738 or 0.625 μM MK-8776 for 72 h. Tail moment of each treatment group were normalized to the mean tail moment of the control cells. *n* = 50 cells per cell line and condition. ***P < 0.001

Next, we tested the potencies of ATR inhibitor AZD6738 and CHK1 inhibitor MK-8776 in liver cancer cell lines. We treated a panel of liver cancer cell lines with increasing concentrations of AZD6738 or MK-8776 for 10–14 days in colony formation assays. While the response to these compounds varied across cell lines, the activities of AZD6738 and MK-8776 on the panel of liver cancer cells were very similar (Fig. 2A). Functionally, liver cancer cell lines can be classified as either sensitive (Huh7, HepG2, MHCC97H, and SNU398) or resistant (Huh6, PLC/PRF/5, and SNU449) to ATR or CHK1 inhibitors (Fig. 2A). Comparable results were also obtained using IncuCyte short-term cell proliferation assays (Fig. 2B, C). ATR or CHK1 inhibition induced apoptosis in the sensitive cells (Huh7 and MHCC97H), as indicated by the caspase-3/7 apoptosis assay, but not in the resistant cell lines (SNU449 and PLC/PRF/5) (Fig. 2D and Additional file 2: Fig. S2). To further address the selective effects of AZD6738 or MK-8776 in sensitive cell lines, we assessed γH2AX protein levels, a marker for DNA damage, following treatment with AZD6738 or MK-8776. To ensure that γH2AX comes from accumulated DNA damage but not increased apoptosis, we added a caspase inhibitor Z-VAD-FMK in the medium. After treatment with AZD6738 or MK-8776, the level of γH2AX was clearly induced in the two sensitive liver cancer cells (Huh7 and MHCC97H) as compared to the resistant cell lines (SNU449 and PLC/PRF/5) (Fig. 2E). Comparable results were also obtained using neutral comet assays (Fig. 2F). In addition, we also investigated whether *ATR* and *CHEK1* expression could be potential biomarkers of drug response to ATR or CHK1 inhibitors. We conducted correlation analyses between expression of *ATR/CHEK1* and drug response of AZD6738 (the drug response data of MK-8776 was absent in public datasets). The results suggested that both *ATR* and *CHEK1* expressions were correlated with sensitivities to ATR inhibition by AZD6738. However, only the expression of *CHEK1* had a significant correlation with drug response of AZD6738 (*P* = 0.014), while *ATR* did not have a statistical significance (Additional file 2: Fig. S1d). This result suggests that using a single target gene (i.e. *ATR* or *CHEK1*) for predicting the drug response of the corresponding inhibitor may not be the most appropriate option in this case.

Together, these findings suggest that ATR and CHK1 could represent potential therapeutic targets for liver cancer. However, effective biomarkers of drug response and powerful drug combination strategies are still required for resistant cells.

### CDC7 inhibitor induces DNA replication stress in HCC cells

**Fig. 3:**
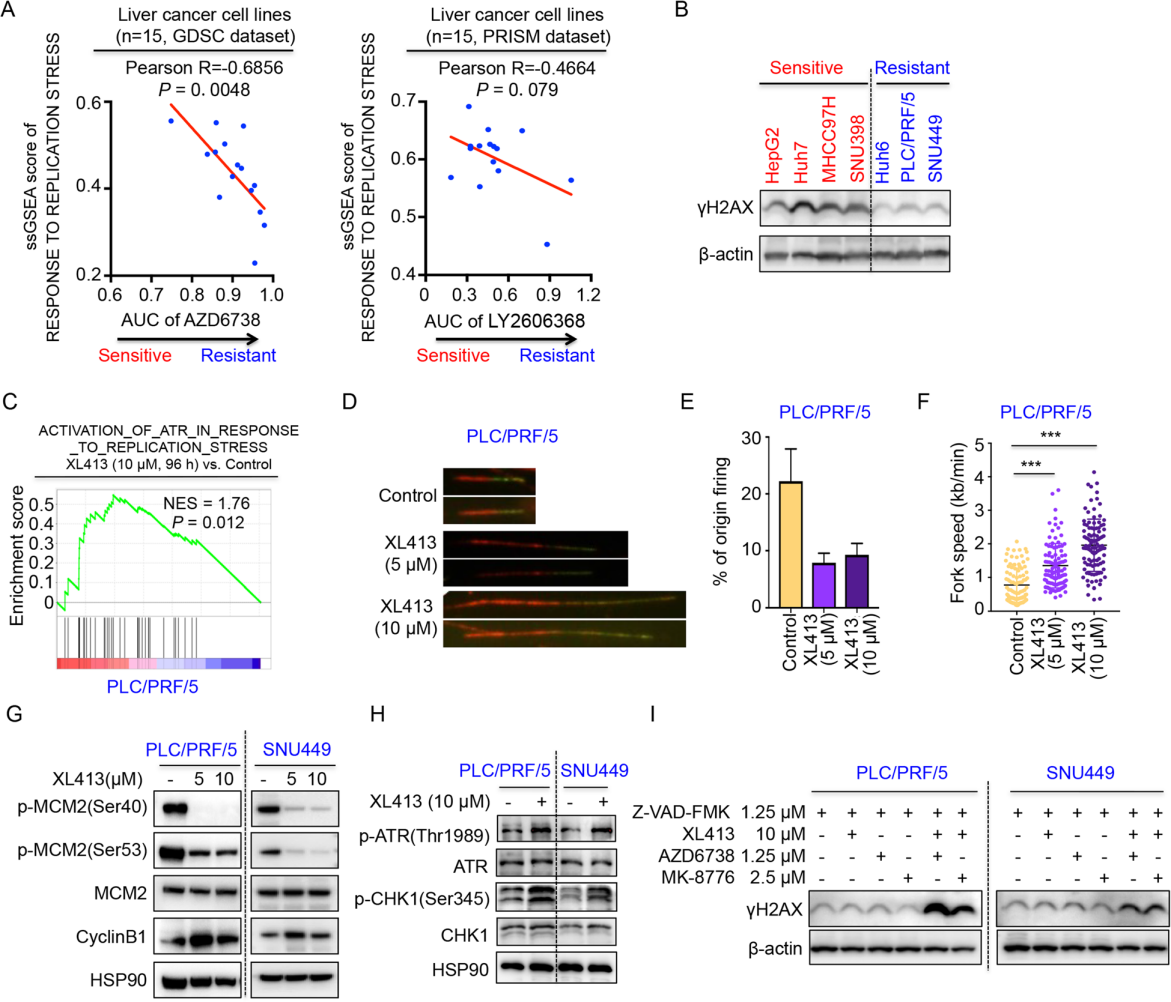
Inhibition of CDC7 induces replication stress in HCC cells. **a** Correlation between the single sample Gene Set Enrichment Analysis (ssGSEA) score of ATR pathway in response to replication stress and the drug sensitivity of AZD6738 (derived from GDSC, left panel) and LY2606368 (derived from PRISM, right panel) in liver cancer cell lines. The *x*-axis depicts the AUC of the indicated drug. Lower values on the *X*-axis imply greater drug sensitivity. **b** Western blot analysis of γH2AX in HCC cell lines sensitive (red) or resistant (blue) to ATR or CHK1 inhibitors. β-actin protein level served as a loading control. **c** GSEA of RNA sequencing data from PLC/PRF/5 cells treated with 10 μM XL413 for 96 h shows that the ATR pathway in response to replication stress was enriched in the presence of XL413. **d** Representative images of ongoing DNA replication tracks observed in PLC/PRF/5 cells cultured in the absence or presence of XL413 with indicated concentrations. **e** Quantification of origin firing in PLC/PRF/5 cells cultured in the absence or presence of XL413 with indicated concentrations. **f** Replication fork speed of PLC/PRF/5 cells was quantified under indicated conditions (*n* > 50 cells per condition). **g** Western blot analysis of MCM2 (Ser40/53) phosphorylation and Cyclin B1 in PLC/PRF/5 and SNU449 cells exposed to the CDC7 inhibitor XL413 (5 μM or 10 μM) for 96 h. HSP90 protein level was used as a loading control. **h** Western blot analysis of ATR (Thy1989) phosphorylation, CHK1 (Ser345) phosphorylation, ATR and CHK1 in PLC/PRF/5 and SNU449 cells exposed to the CDC7 inhibitor XL413 (10 μM) for 24 h. HSP90 protein level was used as a loading control. **i** Western blot analysis of γH2AX as a DNA damage marker in PLC/PRF/5 and SNU449 cells treated with XL413 (10 μM), AZD6738 (1.25 μM), MK-8776 (2.5 μM) or the indicated combinations for 72 h. All cells were treated with the caspase inhibitor Z-VAD-FMK. β-actin protein level served as a loading control

To discover potential biomarkers of response to ATR and CHK1 inhibitors, we performed integrated bioinformatics analyses using gene expression and drug response data from the GDSC and PRISM databases [38, 39]. Enrichment of the replication stress signature was estimated by ssGSEA method based on 37 replication stress-related genes (see the “Methods” section). We found that the abundance of the replication stress signature was significantly associated with the sensitivity to the ATR inhibitor AZD6738 (Fig. 3A, left panel). A similar trend was observed for the relationship between the replication stress signature and the CHK1 inhibitor LY2606368, albeit that this was not significant (LY2606368, Fig. 3A, right panel). Importantly, when we analyzed the pan-cancer cell lines, although the correlations were still statistically significant, the correlation coefficients were relatively low (< 0.15 in AZD6738 and LY2606368, Additional file 2: Fig. S3), indicating that this correlation might be context dependent. Western blot analysis of γH2AX, a marker for both DNA damage and DNA replication stress [18, 48], indicated that the basal levels of γH2AX were clearly higher in four sensitive liver cancer cell lines compared to three cell lines resistant to ATR or CHK1 inhibitors (Fig. 3B).

CDC7 kinase plays important roles in the maintenance of DNA damage response and DNA replication forks and has attracted attention as a target to inducing replication stress [28]. Gene set enrichment analyses of RNA-sequencing data from PLC/PRF/5 cells treated with XL413 showed the enrichment of a gene set related to DNA replication stress (GSE183751, Fig. 3C). To evaluate the effects of the CDC7 inhibitor on origin firing and replication fork speed, we performed DNA fiber assays. PLC/PRF/5 cells treated with XL413 exhibited decreased origin firing as compared to control cells. In contrast, treatment with XL413 led to increased DNA replication fork progression, probably as a compensation for the reduction of origin firing (Fig. 3D-F), which is consistent with previous reports of several different CDC7 inhibitors [28, 49]. When we inhibited CDC7 in liver cancer cells with the CDC7 inhibitor XL413, we observed strong suppression of its downstream target p-MCM2 at XL413 concentrations of 5 μM to 10 μM (Fig. 3G). Western blot analysis revealed that treatment with XL413 caused accumulation of cyclin B1, suggesting an increase in the number of S-G2 phase arrested cells (Fig. 3G). The detection of phosphorylation of ATR and CHK1 by western blot indicated activation of ATR-CHK1 signaling upon CDC7 inhibition (Fig. 3H), further suggesting the potential dependency of ATR-CHK1 signaling for cell survival upon CDC7 inhibitor treatment.

The activation of DNA replication stress by CDC7 inhibition prompted us to study the combination effect of CDC7 and ATR inhibition on DNA damage induction. PLC/PRF/5 and SNU449 cells, which are resistant to ATR and CHK1 inhibitors, were incubated with XL413, AZD6738, and MK-8776 or the indicated combinations for 72 h, after which DNA damage induction was measured. The caspase inhibitor Z-VAD-FMK was also added into the medium to exclude apoptosis-associated γH2AX. We observed synergistic induction of DNA damage by CDC7 and ATR-CHK1 inhibition in both cell lines as demonstrated by immunoblot of γH2AX (Fig. 3I). These findings indicate that DNA replication stress signature could be an effective biomarker for drug response of ATR and CHK1 inhibitors for liver cancer, which can also be exploited for synergistic induction of DNA damage.

### CDC7 inhibition is synergistic with ATR and CHK1 inhibitors in liver cancer cells

**Fig. 4:**
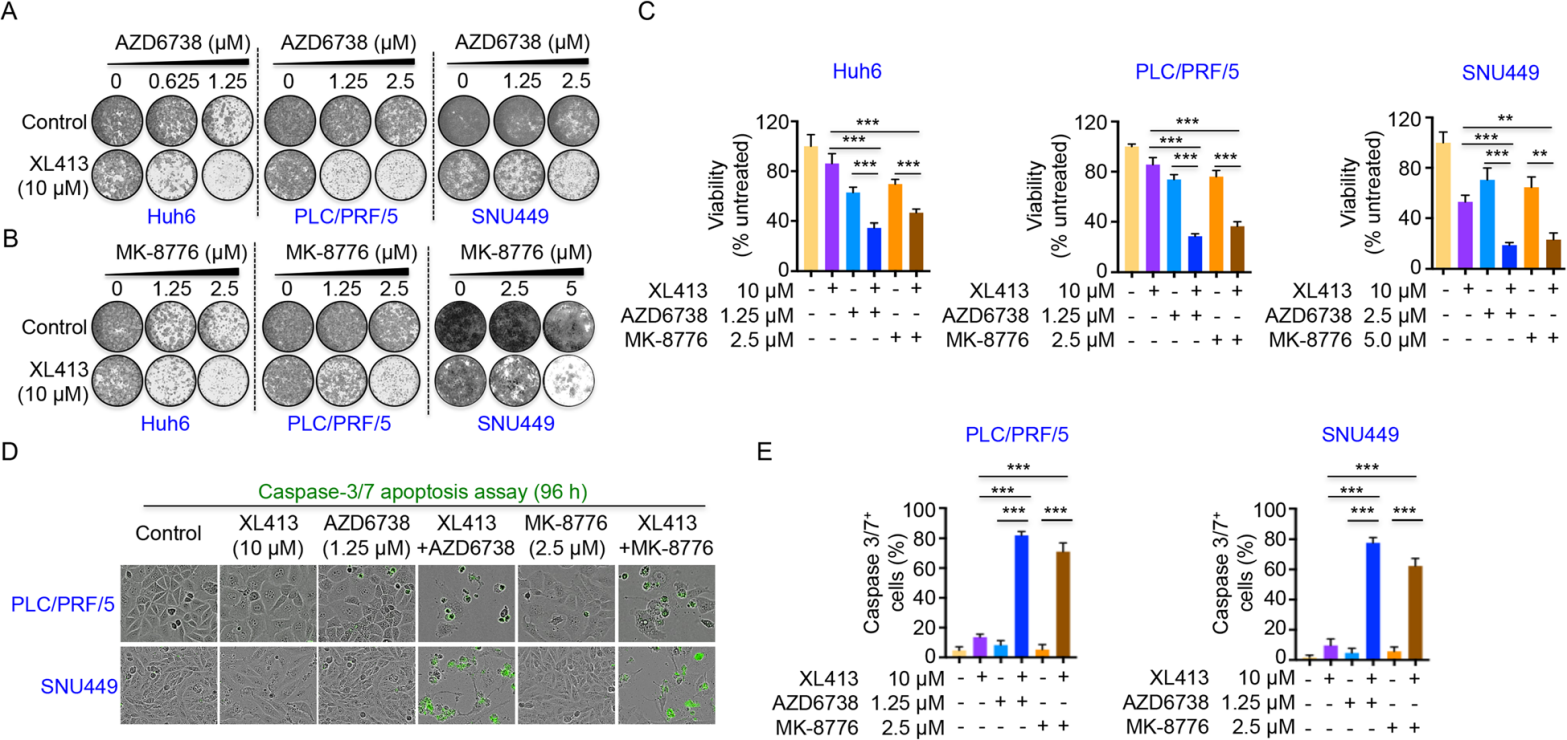
CDC7 inhibitor synergies with AZD6738 or MK-8776 in HCC cells. **a**, **b** Short-term colony formation assays showing synergistic response to XL413 (10 μM) combined with AZD6738 or MK-8776 in three HCC cell lines (resistant to ATR or CHK1 inhibitors: Huh6, PLC/PRF/5 and SNU449) after 4–5 days treatment. **c** Quantification of CellTiter-Blue viability assays of Huh6, PLC/PRF/5, and SNU449 cells treated with XL413, AZD6738, MK-8776 or the combination at the indicated concentrations. **d**, **e** Representative images of PLC/PRF/5 and SNU449 cells treated with XL413, AZD6738, MK-8776 or the indicated combinations for 96 h in the presence of a green fluorescent caspase-3/7 activatable dye. The proportion of cells containing caspase-3/7 staining is shown

The strong synergistic induction of DNA damage by the combination treatment of CDC7 inhibitor with ATR or CHK1 inhibitors led us to explore the synthetic lethal effects of these drug combinations on cell killing. We observed synergistic effects on cell proliferation by XL413 and ATR or CHK1 inhibition (AZD6738 or MK-8776) in all three ATR or CHK1 inhibitor resistant liver cancer cell lines (Fig. 4A–C). Strong synergistic induction of apoptosis was also observed in PLC/PRF/5 and SNU449 cells when XL413 was combined with AZD6738 or MK-8776 as indicated by the IncuCyte caspase-3/7 apoptosis assay (Fig. 4D, E). To further validate the synthetic lethal effects of CDC7 and ATR-CHK1 inhibition, we studied combinations of different CDC7 inhibitors (LY3177833 and TAK-931) and another ATR inhibitor (BAY-1895344) or CHK1 inhibitor (LY2606368) in HCC cell lines (Additional file 2: Fig. S4a-d and Additional file 2: Fig. S5a-d). All these different combinations of CDC7 inhibitors with ATR or CHK1 inhibitors showed striking synergistic responses on proliferation inhibition and apoptosis induction in PLC/PRF/5 and SNU449 cells (Additional file 2: Fig. S4a-d and Additional file 2: Figure S5a-d). To further confirm that induction of DNA replication stress can sensitize cells to ATR or CHK1 inhibitors, we treated cells with cisplatin, a commonly used chemotherapy drug, which induces strong DNA replication stress [50]. Similar to CDC7 inhibition, treatment with cisplatin synthesizes liver cancer cells to AZD6738, further supporting the relation between DNA replication stress and the sensitivity of ATR or CHK1 inhibitors (Additional file 2: Fig. S6a-e).

**Fig. 5:**
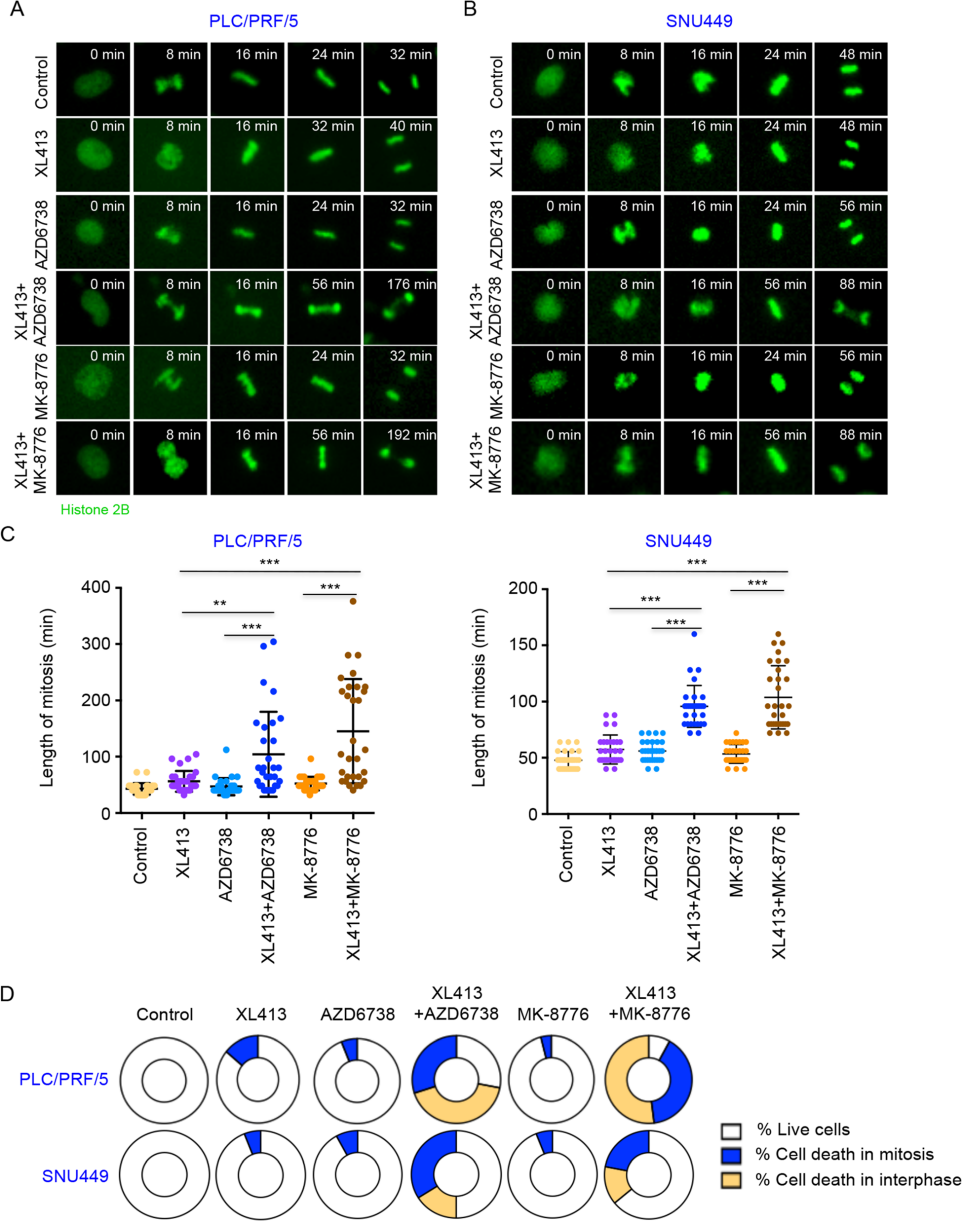
Combined CDC7 and ATR-CHK1 inhibition leads to defective cell cycle of HCC cells. **a**, **b** Representative live cell images of PLC/PRF/5 and SNU449 cells expressing GFP-Histone 2B. Cells treated with XL413 (10 μM), AZD6738 (1.25 μM), MK-8776 (2.5 μM) or the indicated combinations were monitored by time-lapse microscopy. **c** Quantification of mitosis duration based on time-lapse microscopy analysis (*n* > 30 cells per cell line and condition). **d** Percentages of cell fates of PLC/PRF/5 and SNU449 cells in the absence or presence of XL413 (10 μM), AZD6738 (1.25 μM), MK-8776 (2.5 μM) or the indicated combination were analyzed using time-lapse microscopy (*n* = 50 cells per group). **P < 0.01 and ***P < 0.001

Next, we used live cell microscopy to monitor individual cell fates to understand the cell cycle response of liver cancer cells to drug treatment. PLC/PRF/5 and SNU449 cells stably expressing GFP-histone 2B enabled us to track the fates of individual cells and their progenies (Fig. 5A, B). PLC/PRF/5 and SNU449 cells treated with a combination of CDC7 and ATR or CHK1 inhibitors showed significant elevated mitosis duration by comparison to mono-treatment of CDC7 inhibitor or ATR-CHK1 inhibition (Fig. 5C). Furthermore, upon the combination treatment, cell death was also obviously increased in both mitosis and interphase (Fig. 5D). The synergy between ATR-CHK1 inhibition and CDC7 inhibition possibly derives from problems in mitosis (elevated mitosis length and abnormal chromosome segregation) inducing mitotic catastrophe.

### CDC7 inhibition sensitizes HCC cells towards AZD6738 treatment in vivo

**Fig. 6:**
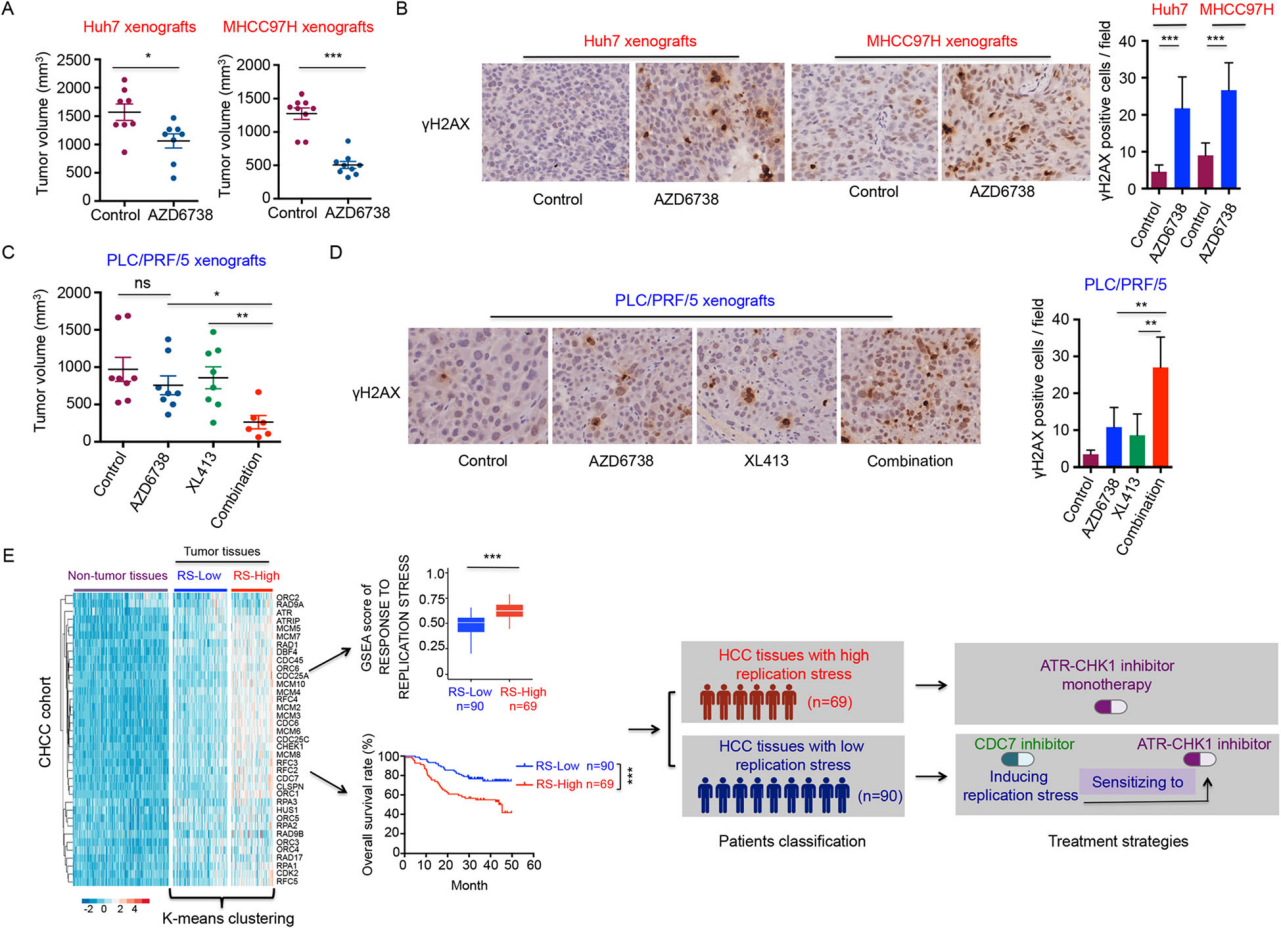
Effects of AZD6738 monotherapy or combined with CDC7 inhibitor in vivo. **a** Tumor volumes of Huh7 and MHCC97H tumor xenografts in BALB/c nude mice following vehicle or AZD6738 (50 mg/kg) treatment for 12 days (Huh7 xenograft) and 18 days (MHCC97H xenograft), respectively. **b** γH2AX staining was performed on formalin-fixed paraffin-embedded Huh7 and MHCC97H xenografts after 12 days (Huh7 xenograft) and 18 days (MHCC97H xenograft) of treatment with vehicle or AZD6738. **c** Tumor volumes of PLC/PRF/5 tumor xenografts in BALB/c nude mice following vehicle, AZD6738 (50 mg/kg), XL413 (50 mg/kg) or combination treatment for 12 days. **d** γH2AX staining was performed on formalin-fixed paraffin-embedded PLC/PRF/5 xenografts after 12 days treatment with vehicle, AZD6738, XL413 or combination. **e** Potential classification and treatment strategies for HCC patients. Upregulation of genes implicated in response to replication stress could serve as a biomarker for the classification of HCC patients. The heatmap shows expression level of genes implicated in replication stress of tumor and adjacent tissues. Among 159 patients with both tumor and paired adjacent tissues available, 69 patients have HCC tissues showing high replication stress, while the remaining 90 patients have HCC tissues with low replication stress according to K-means clustering. Kaplan-Meier curves depicting that high replication stress in tumor tissues correlates with poor prognosis of the patients. Patients with HCC tumors showing high replication stress would potentially benefit from ATR or CHK1 inhibition only, whereas patients with tumors that show low replication stress would need CDC7 inhibition to induce replication stress and sensitize tumor cells to ATR or CHK1 inhibition. *P < 0.05, **P < 0.01 and ***P < 0.001A

To assess whether our in vitro findings can be recapitulated in vivo, we generated Huh7, MHCC97H, and PLC/PRF/5 xenografts. Upon tumor establishment, Huh7 and MHCC97H xenografts were treated with either vehicle or AZD6738 for 12 days and 18 days, respectively. AZD6738 treatment significantly impaired tumor growth of Huh7 and MHCC97H xenografts (Fig. 6A). AZD6738-treated tumors showed increased γH2AX positive cells as compared to tumors treated with vehicle, which indicates that inhibition of ATR induces DNA damage in Huh7 and MHCC97H xenografts (Fig. 6B). Consistent with in vitro results, mice bearing PLC/PRF/5 xenografts that received AZD6738 monotherapy showed a modest reduction in tumor volume, whereas treatment with XL413 combined with AZD6738 significantly reduced tumor burden compared to monotherapy (Fig. 6C). We observed that ATR or CDC7 inhibition led to increased DNA damage in tumor cells, as measured by γH2AX staining. Importantly, combination treatment synergistically induced far stronger DNA damage in tumor tissues (Fig. 6D). In addition, some side effects, such as weight loss and intestinal impairment, have been observed in mice treated with combination of CDC7 plus ATR inhibitors.

### Potential classification and treatment strategy for HCC patients

To investigate whether differences in replication stress levels are also present in clinical samples, we reanalyzed our previous RNA sequencing data, consisting of paired tumor and nontumor tissues from 159 primary HCC patients who underwent curative resection between 2010 and 2014 at Zhongshan Hospital (CHCC cohort) [35]. Based on a set of 37 curated genes involved in response to replication stress, we performed K-means clustering and thereby divided 159 tumors into two subgroups having distinct levels of replication stress-associated gene expression (Fig. 6E). GSEA-based enrichment scores of the replication stress response signature calculated according to the differential fold change values between paired tumor and non-tumor tissues also showed a significant difference between these two subgroups, further supporting the presence of differences in replication stress among clinical samples (Fig. 6E). Notably, survival analysis indicated that tumors of patients having higher levels of transcription of replication stress signature genes experienced worse prognosis, extending the potential utility of this signature from a predictive biomarker to a predictive/prognostic biomarker (Fig. 6E). All above results have also been validated using an external cohort GSE14520 (Additional file 2: Fig. S7a). We also investigated the relationship between replication stress signature and clinical features. Therefore, we used the CHCC cohort, since it has the most comprehensive clinical information. We observed that a high replication stress signature was closely related to the presence of tumor thrombus, high AFP level, advanced BCLC stage, and advanced TNM stage (Additional file 2: Fig. S7b).

According to the findings reported above, a potential treatment strategy for HCC can be envisioned. Patients with a high replication stress gene signature may benefit from monotherapies of ATR or CHK1 inhibitors, while a combination strategy that includes CDC7 inhibition may be required for patients with tumors having low levels of replication stress (Fig. 6E).

## Discussion

In this study, through integrating the data from genome-wide CRISPR screening and analyses of clinical cohorts, we identified ATR and CHK1 as potential therapeutic targets for treatment of HCC. Exploiting CRISPR functional screens to investigate the vulnerabilities of cancer cells can uncover new therapeutic opportunities. Previous large-scale CRISPR screening data from the cancer dependency map portal (depmap.org) provided a treasure trove which has not been explored fully yet. Utilizing this resource, some studies have obtained encouraging findings. For instance, the WRN helicase was identified as a vulnerability of microsatellite unstable cancers [51, 52]. In MYCN-amplified neuroblastoma, 147 selective candidate gene dependencies were identified, providing potential therapeutic insight for this difficult to treat childhood cancers [53]. Interestingly, although CRISPR screening data from DepMap as well as our own screens indicate that all liver cancer cell lines have a dependency on ATR and CHK1, only a subset of these cell lines were responded to ATR or CHK1 inhibitors, presumably due to the discrepancy between complete CRISPR gene knockout and partial small-molecule protein inhibition [54]. Based on this finding, further investigations were undertaken to identify biomarkers of response to these drugs and to identify drug combination strategies to overcome intrinsic resistance to ATR or CHK1 inhibitors.

Almost all currently approved therapies for HCC are based on the “one fits all” principle and consequently fail to deliver significant clinical benefit in an unselected patient population. Thus, for any drug, it is important to identify suitable predictive biomarkers for patient selection. Previously, several studies have reported that multi-gene transcriptome signatures exhibited good performance for predicting clinical efficacy of chemotherapy or targeted therapy [55, 56]. The MammaPrint™ 70-gene signatures and OncotypeDx tests are examples of clinically used gene signatures for the prediction of chemotherapy benefit in early breast cancer [57, 58]. In our study, a gene signature of replication stress was identified, which could not only predict the efficacy of ATR or CHK1 inhibitors but also could be used to identify patients that may benefit from combination therapy with a CDC7 inhibitor.

Our data indicate that the levels of replication stress determine the sensitivity of liver cancer cells to ATR or CHK1 inhibitors. Consistent with this notion, we found that this replication stress signature was also enriched in HCC cells in the presence of CDC7 inhibitor and that CDC7 inhibition sensitizes to ATR and CHK1 inhibition. Previous studies reported that the ATR-CHK1 pathway was required to cope with the high levels of replication stress in cancer cells [12, 29, 59]. Mechanistically, we show that dual CDC7 and ATR or CDC7 and CHK1 inhibition results in an increase in time spent in mitosis and mitotic catastrophe. Generally, replication stress results in stalling of replication forks and is followed by accumulation of single-stranded DNA (ssDNA), which then activates ATR signaling pathway and thus results in phosphorylation of the CHK1 kinase. In our study, XL413 treatment did not completely inhibit fork firing. The small number of active forks was supported by the increased speed of fork progression as a compensation. The activity of the ATR-CHK1 pathway has crucial roles in stabilizing stalled forks and promoting recovery, as well as activating cell cycle checkpoints to prevent cells with un-replicated DNA entering mitosis [60]. In the absence of ATR and CHK1, stalled forks can collapse, and the formation of DNA double-strand breaks (DSBs) will ensue. When entering mitosis, these defective cells are eliminated by mitotic catastrophe [59, 61]. Therefore, intact ATR-CHK1 response is required for the survival of cells experiencing high levels of replication stress, and this pathway can thus be exploited as an attractive therapeutic target for selective elimination of malignant cells.

HCC is a highly aggressive cancer type that lacks effective therapeutics. The most frequent mutations in HCC are currently undruggable, which limits the development of targeted therapies for HCC. It is therefore urgent to develop novel targeted therapies for HCC. Even though a number of ATR and CHK1 inhibitors have been developed and tested as potential anti-tumor agents, the efforts are mainly focused on tumor types exhibiting high levels of replication stress, such as melanoma, pancreatic cancer, and neuroblastoma [14, 62, 63]. In the field of liver cancer, despite some preliminary findings, the therapeutic roles of ATR or CHK1 inhibitors remain to be explored [21, 23]. In this study, we have uncovered potential therapeutic uses of ATR or CHK1 inhibitors in HCC as well as the mechanism of intrinsic resistance to ATR-CHK1 inhibition. The identification of replication stress as a biomarker of response to ATR or CHK1 inhibitors allows selection of patients for monotherapies. Moreover, for patients with low replication stress tumors, addition of CDC7 inhibitor will possibly result in increased replication stress and thereby sensitize tumor cells to ATR or CHK1 inhibitors. With both ATR and CDC7 inhibitors now in clinical trials, our work provides a mechanistic underpinning for their combination [12, 24]. However, some issues still need to be solved. One of the most important problems is toxicity. During our in vivo experiments, we observed that combination of CDC7 and ATR inhibitors caused side effects including weight loss and intestinal impairment in mice. More work is needed to investigate how to best combine these drugs. Sequential or alternating dosing schedules could for instance be used to reduce the toxicity of drugs. That sequential therapies of otherwise toxic drug combinations can be effective was recently shown for the combination of PARP and WEE1 inhibitors [64].

## Conclusions

Our data highlights the potential of targeting ATR-CHK1 signaling, either alone or in combination with CDC7 inhibition, for the treatment of liver cancer based on the level of replication stress. Our results demonstrate the feasibility of personalized therapeutic opportunities in liver cancer.

## Supplementary Information

**Additional file 1: Table S1.** Kinome screen data for HCC cells.

**Additional file 2: Fig S1.** ATR and CHK1 are potential therapeutic targets for HCC. **Fig S2.** Effects of ATR and CHK1 inhibitors on apoptosis induction of HCC cells. **Fig S3.** Relationship between the replication stress response signature and drug response. **Fig S4.** CDC7 inhibitors synergies with ATR or CHK1 inhibition in HCC cells. **Fig S5.** CDC7 inhibition synergies with ATR or CHK1 inhibitors in HCC cells. **Fig S6.** Cisplatin synergies with ATR inhibitor in HCC cells. **Fig S7.** Clinical association of replication stress signature.

**Additional file 3: Table S2.** List of 220 DNA repair genes.

**Additional file 4.** Original blots for all western blots results.

## Data Availability

Processed and raw sequencing data can be downloaded from NCBI GEO (GSE183751): https://www.ncbi.nlm.nih.gov/geo/query/acc.cgi?acc=GSE183751 [65]. Original blots for all western blots shown are provided in Additional file 4.
