# Supplementary material for: Targeting CDC7 potentiates ATR-CHK1 signaling inhibition through induction of DNA replication stress in liver cancer
Source: Genome Med. 2021 Oct 18;13:166. doi: 10.1186/s13073-021-00981-0 (PMC8524847; doi:10.1186/s13073-021-00981-0)
Supplement: Supplementary file 2 — Additional file 2: Fig S1. ATR and CHK1 are potential therapeutic targets for HCC. Fig S2. Effects of ATR and CHK1 inhibitors on apoptosis induction of HCC cells. Fig S3. Relationship between the replication stress response signature and drug response. Fig S4. CDC7 inhibitors synergies with ATR or CHK1 inhibition in HCC cells. Fig S5. CDC7 inhibition synergies with ATR or CHK1 inhibitors in HCC cells. Fig S6. Cisplatin synergies with ATR inhibitor in HCC cells. Fig S7. Clinical association of replication stress signature. [file 13073_2021_981_MOESM2_ESM.pdf]

Additional file 2

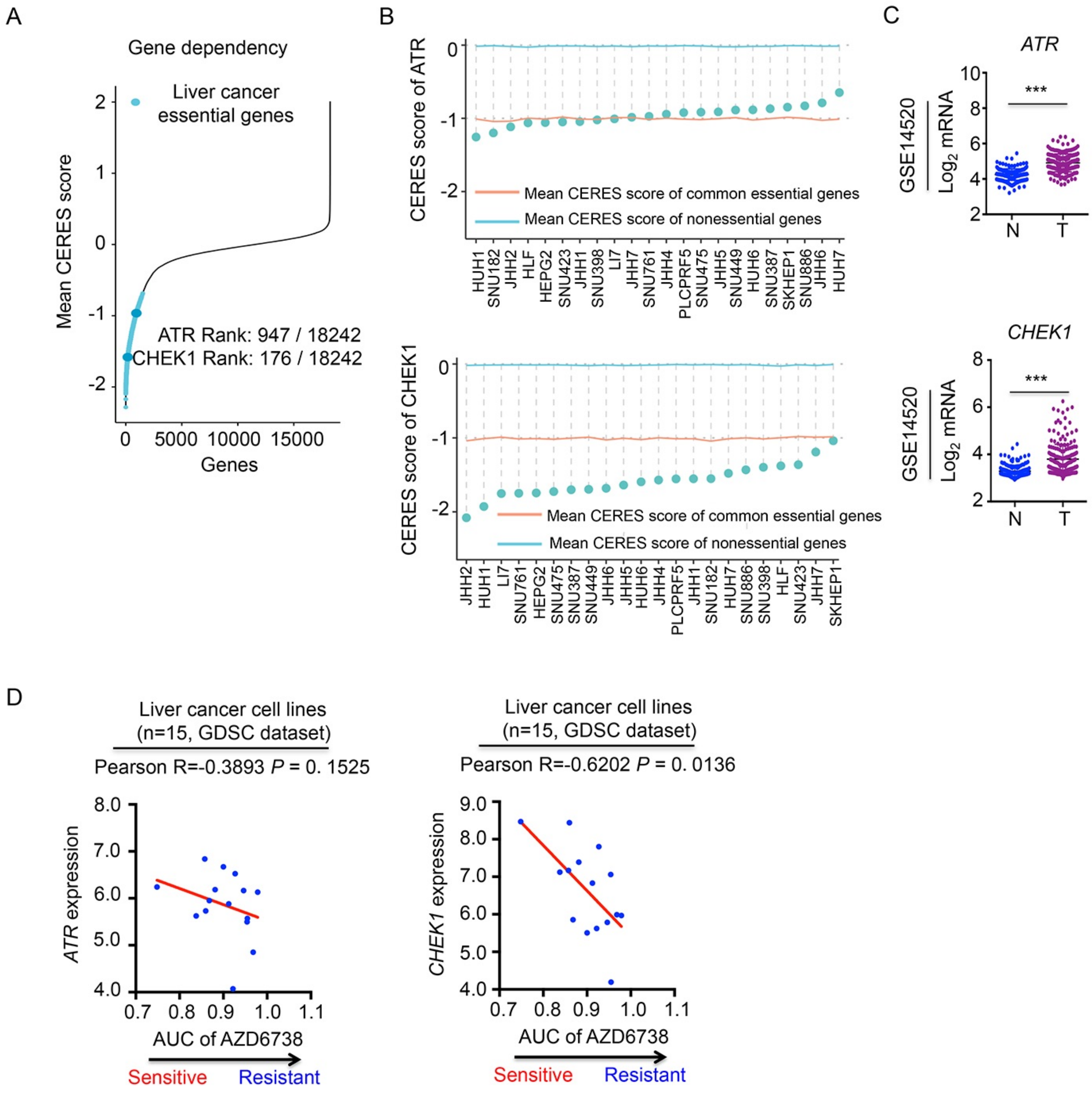

Fig. S1. ATR and CHK1 are potential therapeutic targets for HCC.

a Gene dependency score of liver cancer cell lines analyzed from the Dependency Map database. **b** CERES scores of ATR and CHEK1 in HCC cells derived from DepMap database. **c** mRNA levels of *ATR* and *CHEK1* in tumor tissues and paired non-tumor tissues in the cohort of GSE14520 database (n=213). **d** The correlation between expression of *ATR* or *CHEK1* and response to ATR inhibitor AZD6738. ***$P < 0.001$.

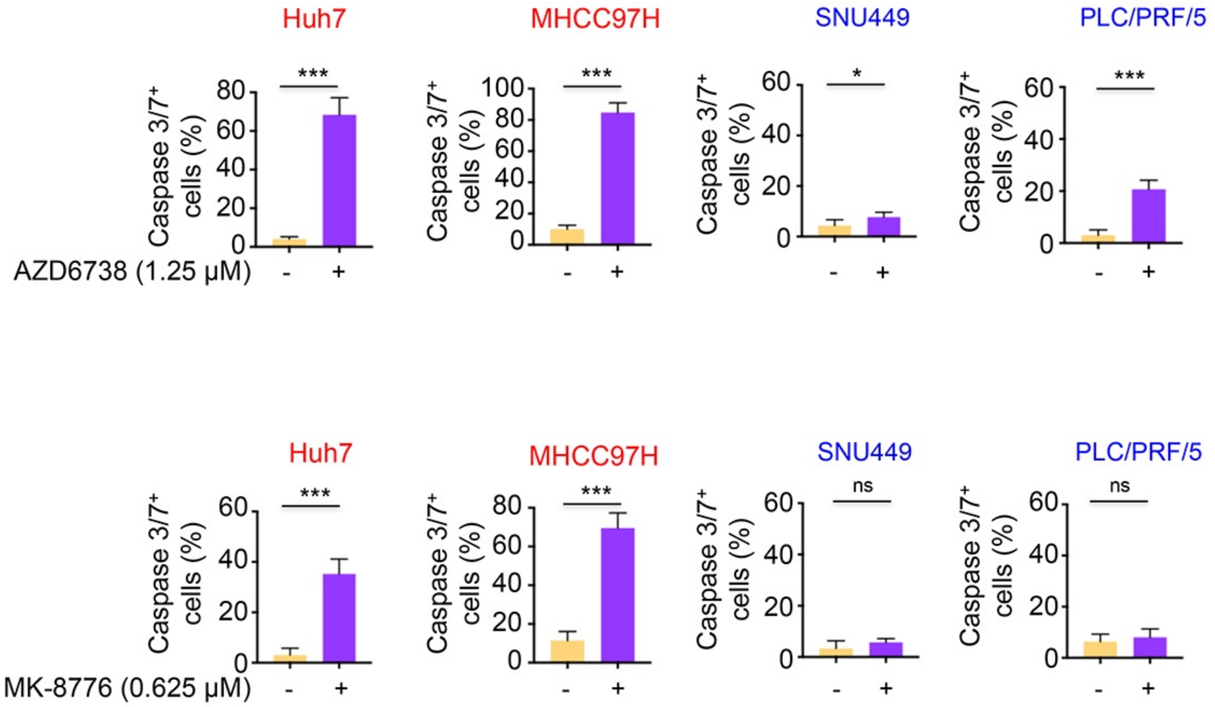

Fig. S2. Effects of ATR and CHK1 inhibitors on apoptosis induction of HCC cells.

HCC cells in the presence or absence of AZD6738, MK-8776 were dyed with a green fluorescent caspase-3/7 activatable reagent. Cell apoptosis was quantified as the proportion of cells containing caspase-3/7. ***$P < 0.001$.

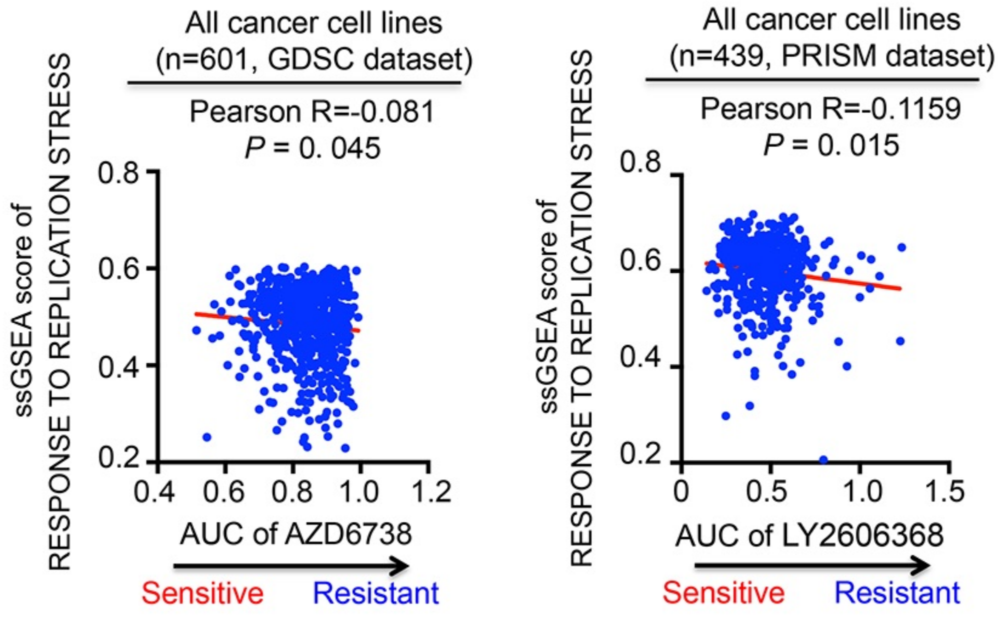

Fig. S3. Relationship between the replication stress response signature and drug response. Correlation between the single sample Gene Set Enrichment Analysis (ssGSEA) score of ATR pathway in response to replication stress and the drug sensitivity of AZD6738 (derived from GDSC, left panel) and LY2606368 (derived from PRISM, right panel) across pan cancer cell lines. The x axis depicts the AUC of the indicated drug. Lower values on the x-axis imply greater drug sensitivity.

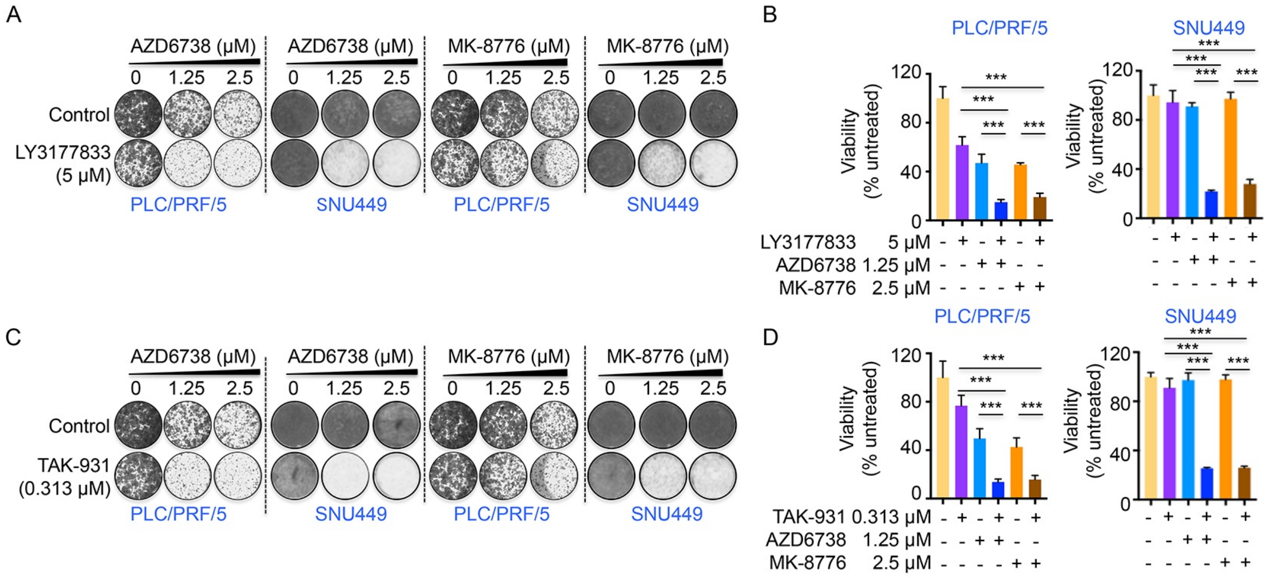

Fig. S4. CDC7 inhibitors synergies with ATR or CHK1 inhibition in HCC cells.

a, b Colony formation assays and quantification of cell viability assays show synergistic response to LY3177833 (CDC7 inhibitor) combined with AZD6738 or MK-8776 in PLC/PRF/5 and SNU449 cells after 4-5 days treatment. **c, d** Colony formation assays and quantification of cell viability assays show synergistic response to TAK-931 (CDC7 inhibitor) combined with AZD6738 or MK-8776 in PLC/PRF/5 and SNU449 cells after 4-5 days treatment. ***P < 0.001.

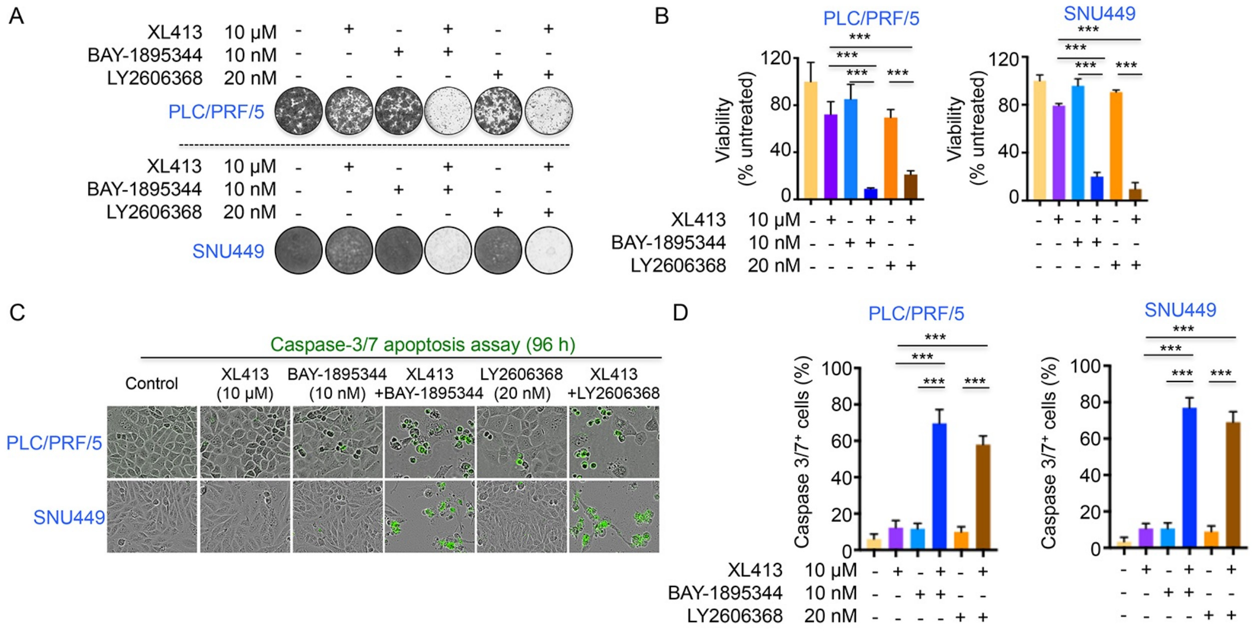

Fig. S5. CDC7 inhibition synergies with ATR or CHK1 inhibitors in HCC cells.

a, b Colony formation assays and quantification of cell viability assays show synergistic response to XL413 (CDC7 inhibitor) combined with BAY-1895344 (ATR inhibitor) or LY2606368 (CHK1 inhibitor) in PLC/PRF/5 and SNU449 cells after 4-5 days treatment. **c, d** Representative images of PLC/PRF/5 and SNU449 cells treated with 10 μM XL413 (CDC7 inhibitor), 10 nM BAY-1895344 (ATR inhibitor), 20nM LY2606368 (CHK1 inhibitor) or the indicated combinations in the presence of a green fluorescent caspase-3/7 activatable dye. Cell apoptosis was quantified as the proportion of cells containing caspase-3/7. ****P* < 0.001.

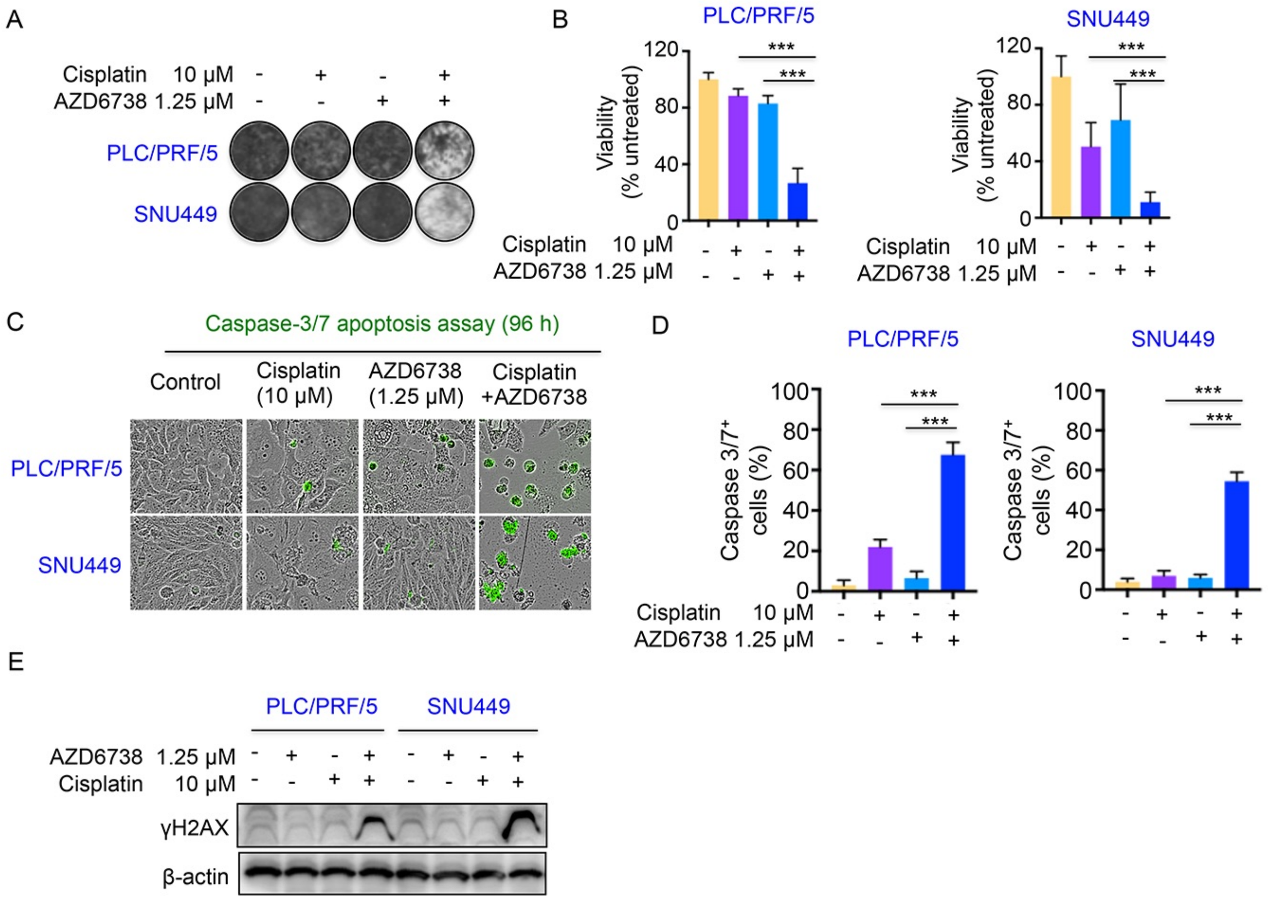

Fig. S6. Cisplatin synergies with ATR inhibitor in HCC cells.

a, b Colony formation assays and quantification of cell viability assays show synergistic response to cisplatin combined with AZD6738 in PLC/PRF/5 and SNU449 cells after 4-5 days treatment. **c** Representative images of PLC/PRF/5 and SNU449 cells treated with AZD6738, cisplatin or the combination at indicated concentration in the presence of a green fluorescent caspase-3/7 activatable dye. **d** Cell apoptosis was quantified as the proportion of cells containing caspase-3/7. **e** Western blot analysis of γH2AX as a DNA damage marker in PLC/PRF/5 and SNU449 cells treated with AZD6738, cisplatin or the combination at indicated concentration for 72 hours. β-actin protein level was served as a loading control. ***P < 0.001.

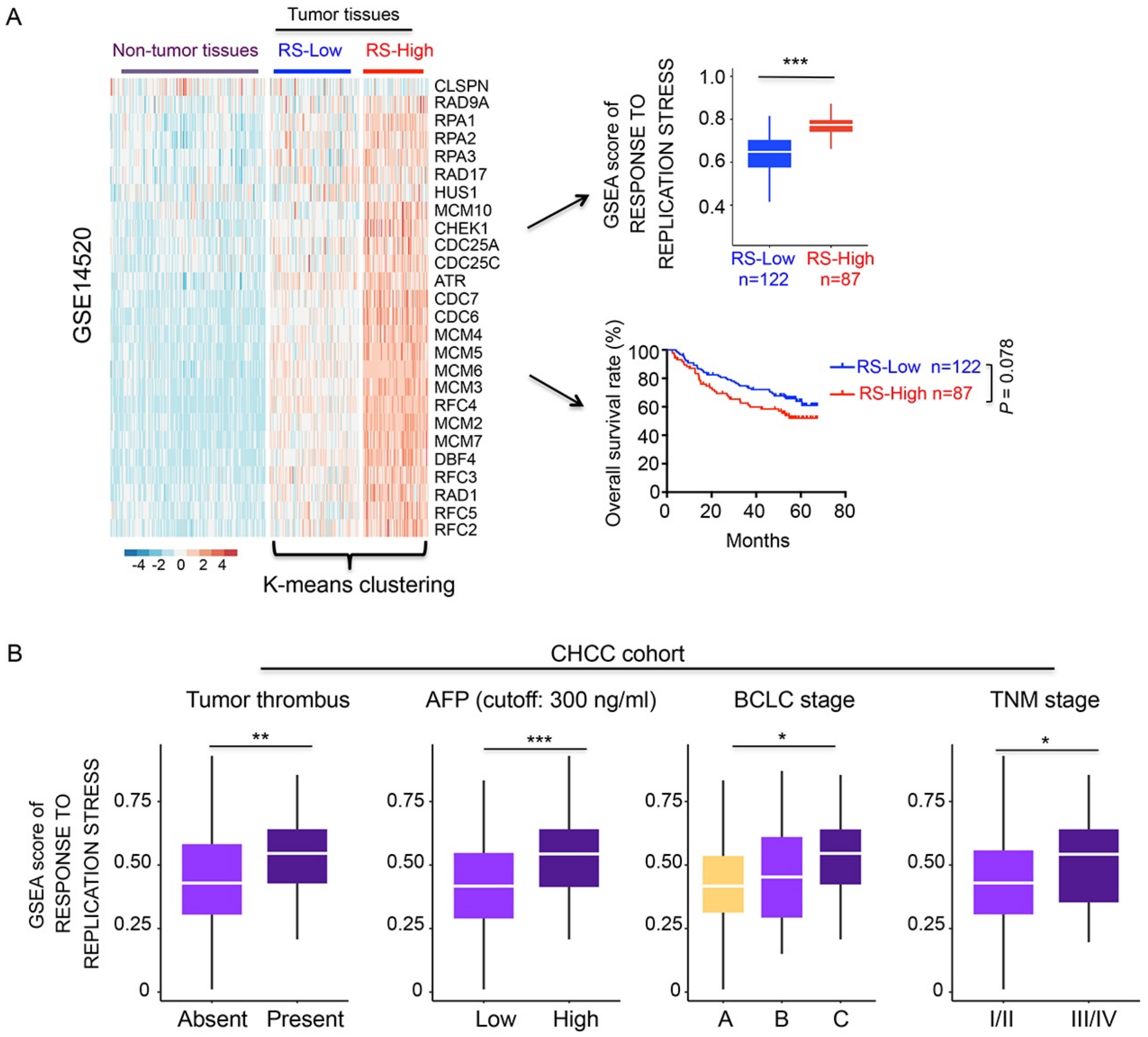

Fig. S7. Clinical association of replication stress signature.

a Validation of prognostic value of replication stress signature in GSE14520 cohort. The heatmap shows expression level of genes implicated in replication stress of tumor and adjacent tissues. Due to the platform limitation, only 26 replication stress-related genes were included in this cohort. Among 213 patients with both tumor and paired adjacent tissues available, 87 patients have HCC tissues showing high replication stress levels, while the remaining 122 patients have HCC tissues with low replication stress levels according to K-means clustering. Kaplan-Meier curves depicting that high replication stress in tumor tissues tends to correlate with poor prognosis of the patients **b** High replication stress is related to several critical clinical features in CHCC cohort, including the presence of tumor thrombus, high AFP level, advanced BCLC stage and advanced TNM stage. $^*P < 0.05$, $^{**}P < 0.01$ and $^{***}P < 0.001$.
