# Supplementary material for: Targeting CDC7 potentiates ATR-CHK1 signaling inhibition through induction of DNA replication stress in liver cancer
Source: Genome Med. 2021 Oct 18;13:166. doi: 10.1186/s13073-021-00981-0 (PMC8524847; doi:10.1186/s13073-021-00981-0)
Supplement: Supplementary file 4 — Additional file 4. Original blots for all western blots results. [file 13073_2021_981_MOESM4_ESM.ppt]

## Slide 1
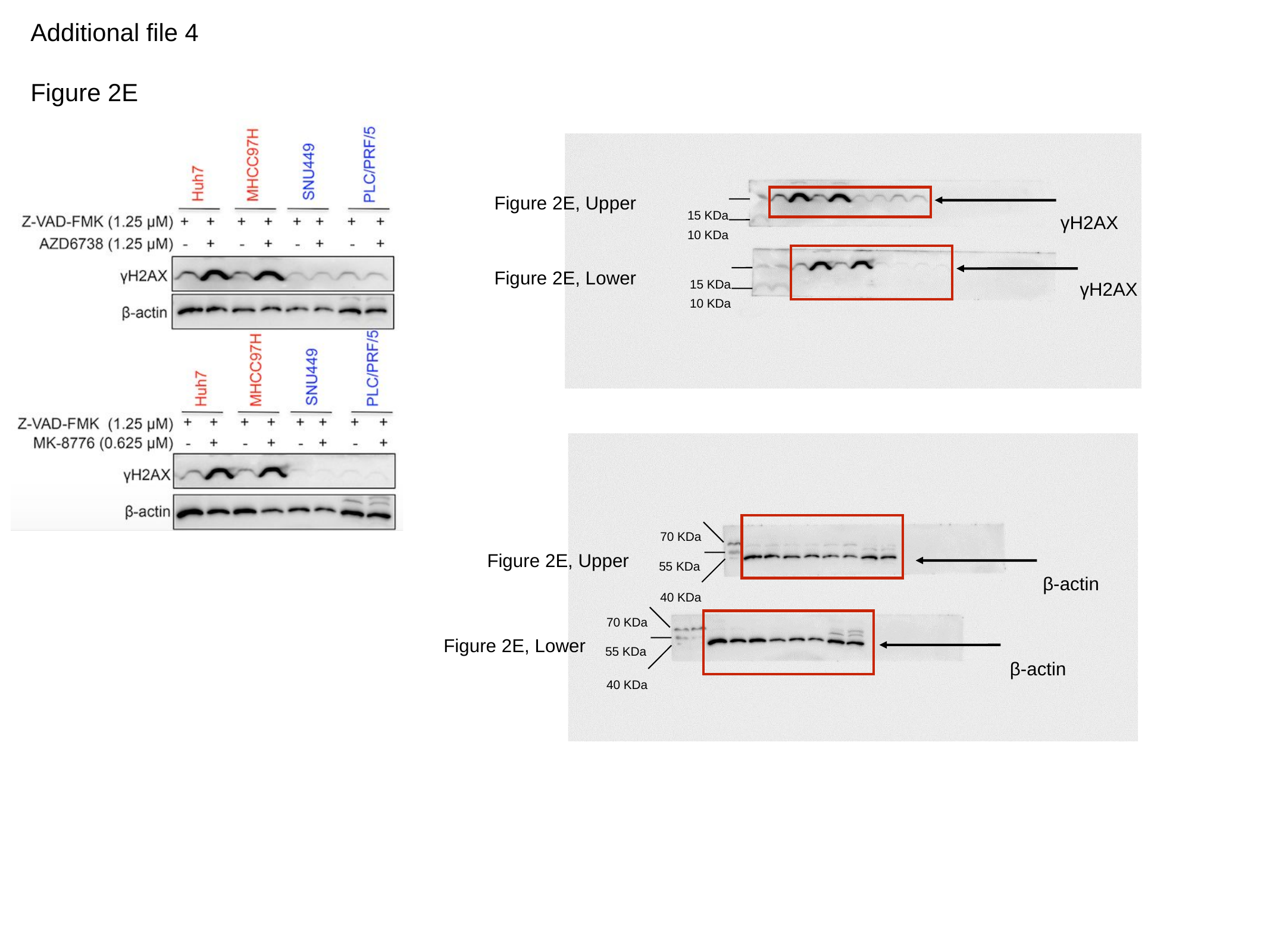

Additional file 4
Figure 2E
γH2AX
15 KDa
Figure 2E, Upper
10 KDa
γH2AX
15 KDa
Figure 2E, Lower
10 KDa
70 KDa
55 KDa
β-actin
Figure 2E, Upper
40 KDa
70 KDa
55 KDa
β-actin
Figure 2E, Lower
40 KDa

## Slide 2
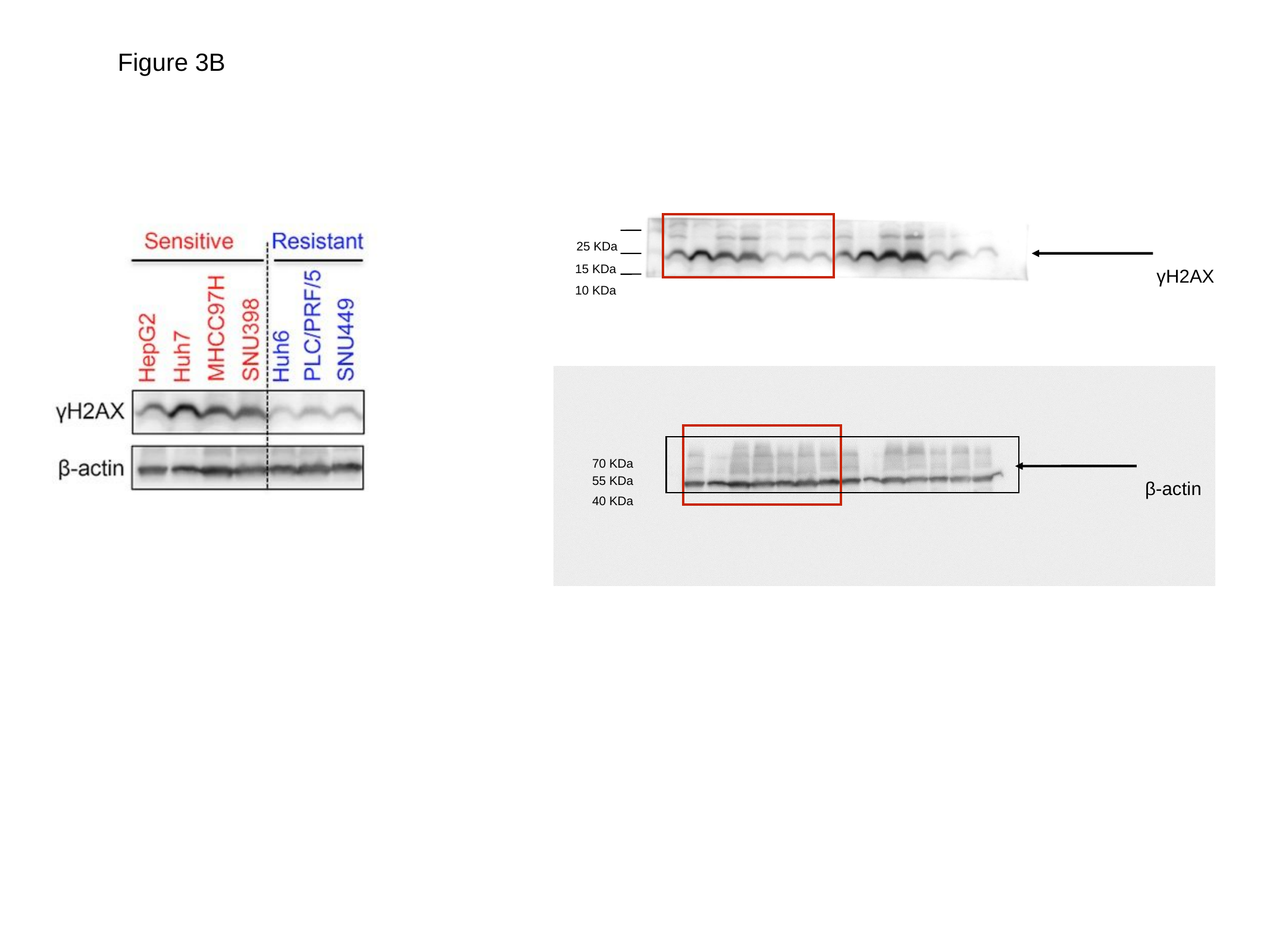

Figure 3B
25 KDa
γH2AX
15 KDa
10 KDa
70 KDa
β-actin
55 KDa
40 KDa

## Slide 3
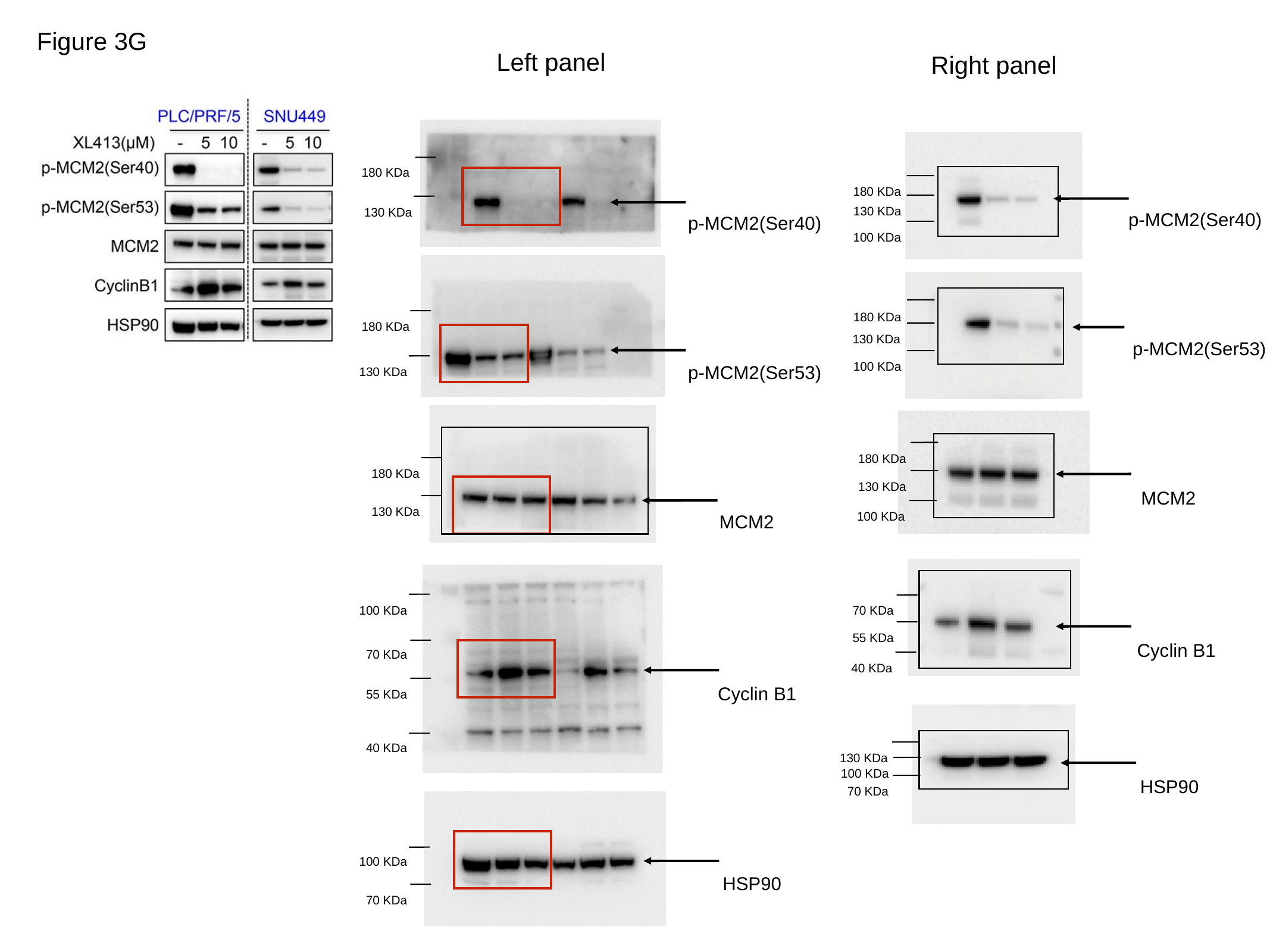

Figure 3G
Left panel
Right panel
180 KDa
180 KDa
p-MCM2(Ser40)
130 KDa
p-MCM2(Ser40)
130 KDa
100 KDa
180 KDa
180 KDa
p-MCM2(Ser53)
130 KDa
p-MCM2(Ser53)
100 KDa
130 KDa
180 KDa
180 KDa
MCM2
130 KDa
MCM2
130 KDa
100 KDa
70 KDa
100 KDa
55 KDa
Cyclin B1
70 KDa
40 KDa
Cyclin B1
55 KDa
40 KDa
130 KDa
100 KDa
HSP90
70 KDa
100 KDa
HSP90
70 KDa

## Slide 4
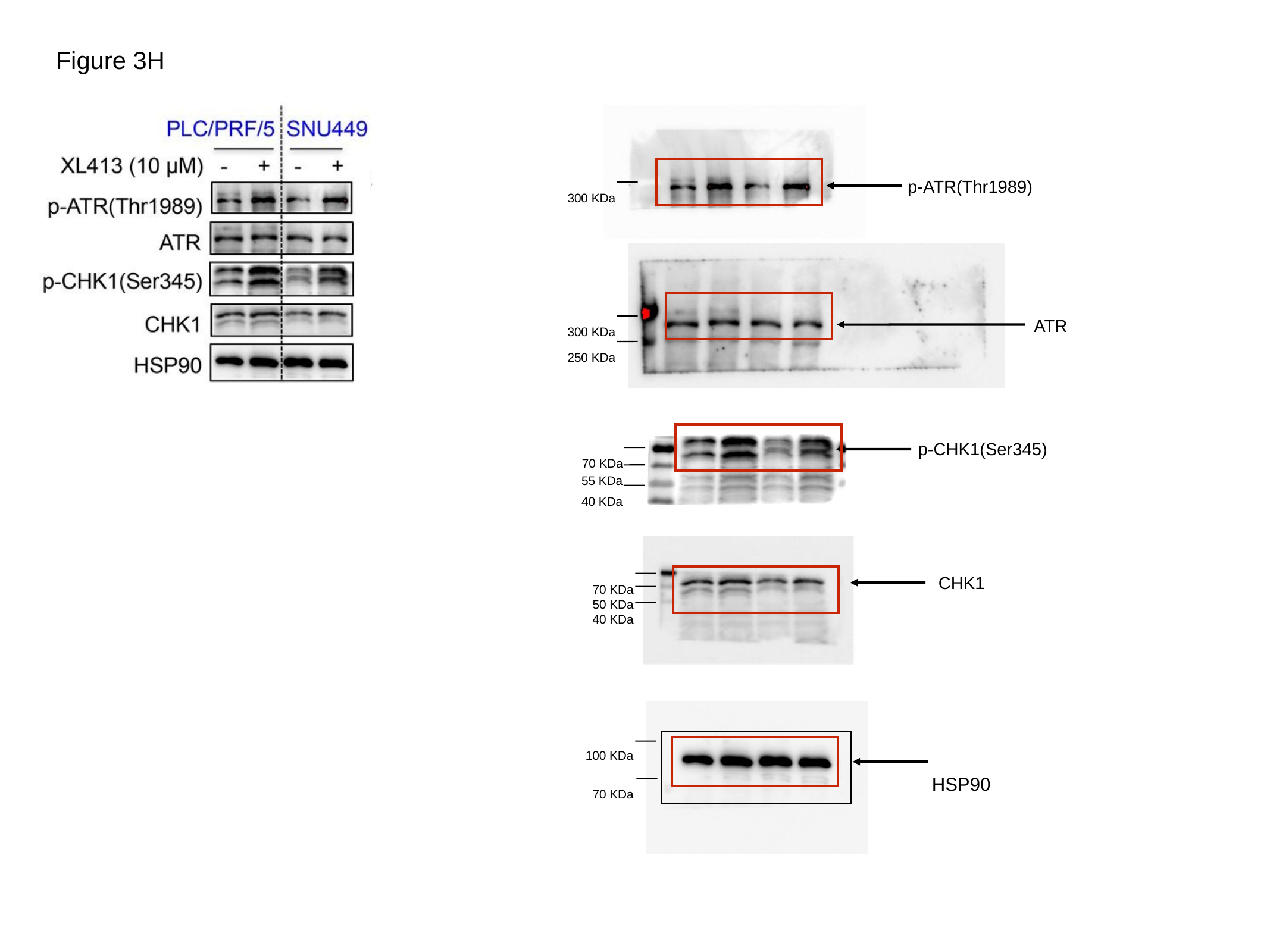

Figure 3H
300 KDa
p-ATR(Thr1989)
300 KDa
ATR
250 KDa
70 KDa
p-CHK1(Ser345)
55 KDa
40 KDa
70 KDa
CHK1
50 KDa
40 KDa
100 KDa
HSP90
70 KDa

## Slide 5
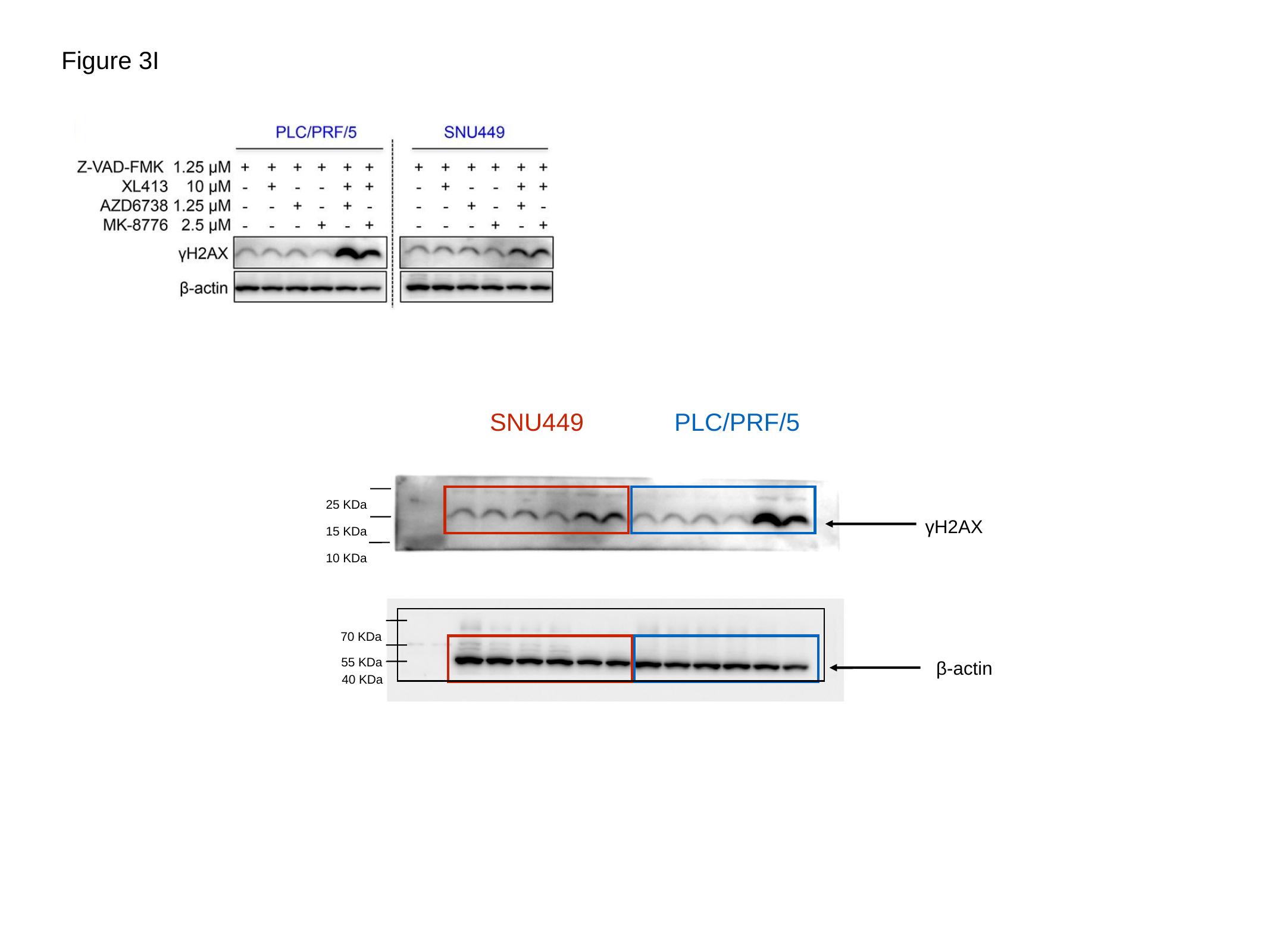

Figure 3I
SNU449
PLC/PRF/5
25 KDa
γH2AX
15 KDa
10 KDa
70 KDa
55 KDa
β-actin
40 KDa

## Slide 6
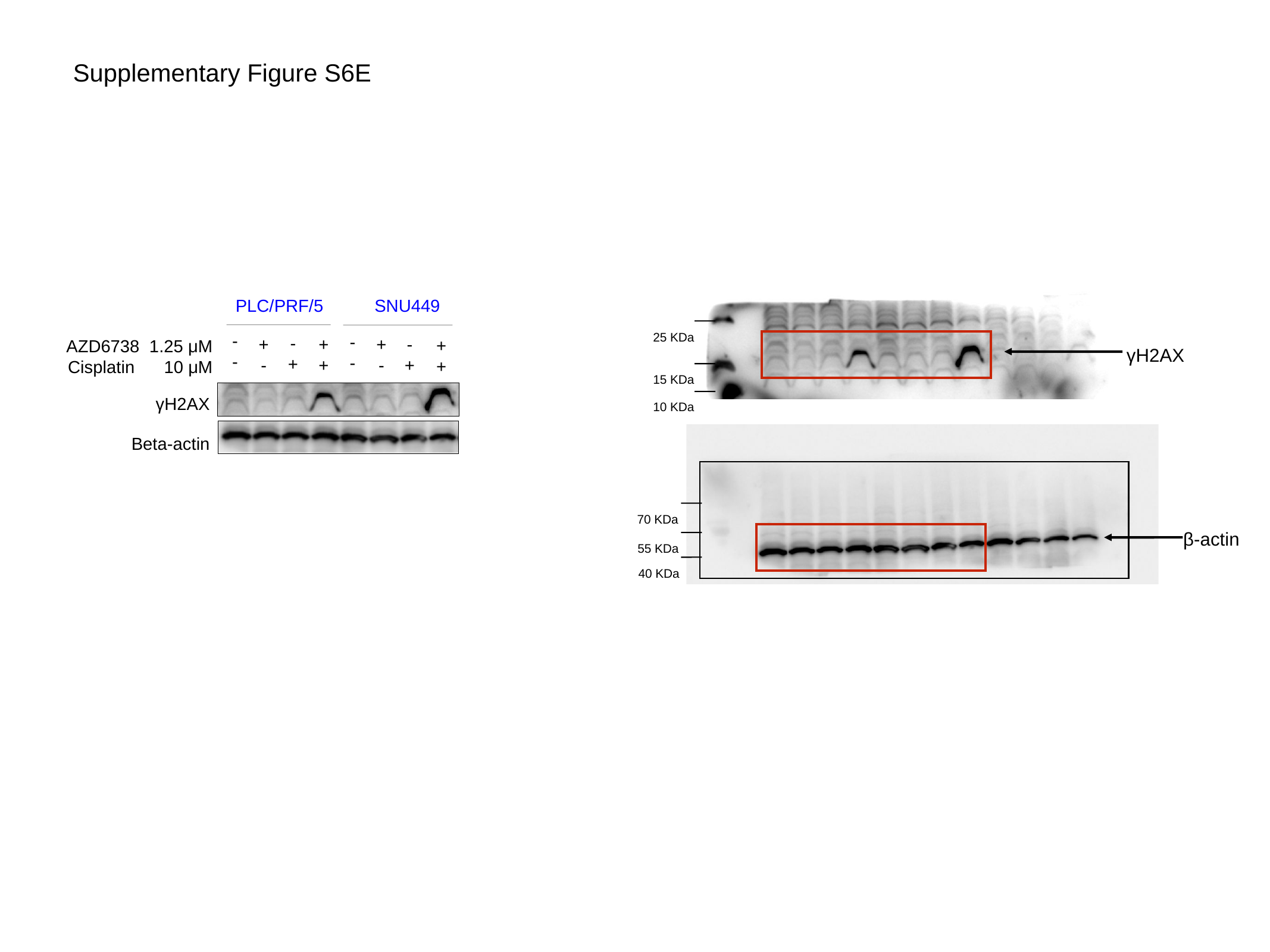

Supplementary Figure S6E
PLC/PRF/5
SNU449
-
-
-
-
25 KDa
AZD6738 1.25 μM
Cisplatin 10 μM
γH2AX
-
+
+
-
-
+
+
-
+
+
+
+
15 KDa
γH2AX
Beta-actin
10 KDa
70 KDa
β-actin
55 KDa
40 KDa
